# ISD-YOLO: A lightweight and efficient feature extraction network for invoice seal detection

**DOI:** 10.1371/journal.pone.0358190

**Published:** 2026-09-11

**Authors:** Xiaosi Song, Xiaohong Zhao, Caihua Ma, Wenpei Xu, Peng Shen

**Affiliations:** 1 Hengshui College of Vocational Technology, Hengshui, China; 2 North China Institute of Aerospace Engineering, Langfang, China; Nanjing Forestry University, CHINA

## Abstract

Invoice seal detection plays an important role in intelligent invoice processing by improving review efficiency and reducing potential financial risks. However, existing methods still face challenges in multi-scale feature representation, complex background interference, and balancing detection accuracy with computational efficiency. In this study, a lightweight detection model, ISD-YOLO, is proposed for invoice seal detection. Specifically, the C3k2-ConvFormer-CGLU module (C3k2_CFC) is designed to enhance multi-scale feature representation by combining local feature enhancement and gated feature selection. A C2BRA module based on bi-level routing attention is introduced to improve the extraction of discriminative seal features by dynamically selecting important regions. In addition, a deformable attention module (DAttention) is incorporated to enhance the focus on key regions and reduce background interference. Furthermore, a lightweight detection head (EfficientHead) is developed to reduce computational complexity while maintaining detection performance, providing potential for resource-constrained applications. Experiments were conducted on a dataset consisting of public seal images and real invoice images. Compared with the baseline model, ISD-YOLO improves mAP50 and mAP50:95 by 2.1% and 2.3%, respectively. Compared with existing detection methods, ISD-YOLO achieves an F1-score of 95.3% and an mAP50 of 97.2%, while requiring only 2.3 M parameters and 5.0 GFLOPs. The results demonstrate that ISD-YOLO achieves a favorable balance between detection accuracy and computational efficiency under the evaluated invoice seal detection conditions. Its lightweight design indicates potential for resource-constrained applications, while further validation under broader scenarios and on actual edge devices is still required.

## 1. Introduction

With the continuous digitalization of fiscal and taxation services and the growing intelligence of document processing, invoices have assumed an increasingly important role in reimbursement and accounting, tax filing, and audit inspection. As a key indicator of an invoice’s authenticity, integrity, and ownership credibility, the seal is a critical element in document compliance review. Automated invoice seal detection can provide an effective basis for document review by identifying anomalies such as missing, incorrect, or forged seals, as well as document tampering. This capability can reduce fiscal and taxation risks and management costs, improve document processing efficiency, and facilitate business process automation [1]. In practical applications, however, invoices typically pass through multiple stages, including generation, sealing, scanning, and archiving, during which seal regions are often affected by occlusion, overlap, blur, fading, noise, illumination variations, and rotation. These factors give rise to complex visual characteristics in seal regions and thereby pose substantial challenges to accurate detection. At the same time, as the scale of document processing continues to expand, traditional manual verification methods can no longer simultaneously guarantee efficiency, accuracy, and consistency, making them inadequate for practical business requirements. Therefore, research on invoice seal detection methods with high precision and strong robustness can not only provide technical support for seal authenticity verification, anomalous document interception, and fiscal and taxation risk control, but also advance the application and development of complex document image understanding and target detection technologies in fiscal and taxation scenarios.

Within this framework, initial research investigated seal identification techniques utilizing conventional feature extraction to facilitate swift detection and classification of invoice seals. For instance, Bao et al. [2] utilized a seal positioning strategy grounded in the RGB color framework. This technique mainly hinged on the premise that seals are typically crimson and identified seal positions via layer segmentation. Su [3] proposed a seal authentication method utilizing the gap between the seal outline and center, plus the edge gray-scale intensity, thereby utilizing the intrinsic characteristics of the seal for verification. Relative to human scrutiny, such techniques provide specific benefits regarding expense, productivity, and precision. Nevertheless, these traditional feature extraction-oriented approaches primarily depend on attributes like hue, dimension, and outline for localization. In intricate and varied practical environments, seal traits are frequently influenced by elements like obstruction, smudging, and interference. Consequently, consistent and precise identification is frequently challenging to attain, resulting in inferior robustness and reduced recognition accuracy.

Owing to the swift evolution of artificial intelligence, deep learning, as a vital subfield within this area, has likewise attained significant breakthroughs. Machine learning-based methods that employ target detection techniques have enabled the automatic recognition of seals in documents, thereby providing an effective solution for invoice seal detection [4]. Target detection methods can identify and classify targets in an image, localize them using bounding boxes, and provide corresponding confidence scores [5]. Such frameworks are generally educated on data collections comprising visual inputs and related labels, where these labels define the positions and types of objects through bounding boxes. Such labels are frequently termed ground-truth data, while the recognition outcomes generated by the system are classified as forecasts.

Localization techniques for specific targets are generally classified into two primary categories: two-stage architectures and single-stage frameworks. Two-stage approaches typically produce proposal bounding boxes during the initial phase, subsequently refining the object’s location and categorizing within the subsequent phase. Notable two-stage architectures encompass R-CNN [6], Faster R-CNN [7], and Mask R-CNN [8]. While these approaches usually deliver superior detection precision, their comparatively slow inference speeds restrict their usability in real-time environments. In contrast, single-stage models directly predict object labels and bounding box parameters through a single forward pass without requiring a separate region proposal step, thereby enabling end-to-end localization with improved computational efficiency. Therefore, single-stage detectors are typically more appropriate for real-time tasks. Particularly, SSD [9] and the YOLO suite [10–13] have attracted substantial attention because of their swift inference times. Crucially, extensive investigations have shown that YOLO outperforms two-stage frameworks like Faster R-CNN and also performs superiorly compared to other single-stage alternatives, including SSD and EfficientDet [14]. Furthermore, YOLO has exhibited superior outcomes in various diverse application settings [15–19]. When contrasted with many contemporary detectors, YOLO provides quicker inference while preserving robust accuracy, rendering it especially appropriate for real-time identification missions. Consequently, YOLO has turned into one of the most prominent and broadly utilized architectures within the domain of object detection.

The YOLO framework, proposed by Joseph Redmon in 2016, was developed to facilitate instantaneous recognition within picture data and video streams. Since its initial release, the YOLO lineage has experienced persistent refinement, producing numerous subsequent iterations. In 2023, Ultralytics launched YOLOv8 [20], which supports various visual applications, encompassing object localization, pixel-level segmentation, and picture categorization. YOLOv10 [21], published in May 2024, eliminated dependence on NMS and alleviated the influence of IoU cutoffs on recognition results. Following this, YOLO11, introduced in October 2024, improved inference velocity while preserving recognition precision via several structural enhancements; YOLOv12 [22] further emphasized attention mechanism-based architectural design to improve real-time detection performance; YOLOv13 [23] augmented the model’s adaptive visual perception capability by incorporating a hypergraph module. YOLO26 [24], launched by Ultralytics in 2026 as a next-generation YOLO model, employs a native end-to-end architecture, eliminates NMS post-processing, and incorporates multiple enhancements to improve small object detection, edge deployment efficiency, and CPU inference performance. Overall, the progression of the YOLO series has consistently advanced the trade-off between inference efficiency and detection robustness, providing effective technical support for invoice seal detection tasks.

Throughout the stages of invoice creation, stamping, scanning, and storage, seal zones frequently encounter issues such as occlusion, overlapping, blurring, discoloration, and lighting fluctuations, which significantly elevate the complexity of reliable detection. To enhance identification precision and fulfill industrial standards, scholars have devised numerous machine learning- and deep learning-oriented strategies for invoice seal localization. For instance, [25] introduced a neural network-driven automatic passport seal classification framework to precisely identify and categorize passport seals. Within [26], a fully convolutional neural network was utilized to partition images into two classes: seal and non-seal. This structure was constructed using the pre-trained VGG-16 model and tested on the StaVer dataset, reaching a precision of 87% and a recall of 84%. Nevertheless, the authors pointed out that this technique still encounters challenges in segmenting black seals. Simultaneously, [27] created a compact neural network architecture, YOLO-Stamp, derived from the YOLO detection structure. Characterized by merely 128,000 parameters, this model reached an accuracy of 90.6% and a recall of 90.7% on the scanned pseudo-official dataset (SPODS). Moreover, [28] proposed a seal extraction process based on a generative adversarial network (GAN) to extract texture features from seal-embedded photographs. Building on this, an improved seal text recognition method, PP-OCR, was further presented to effectively recognize text within seals of various types. Likewise, [29] employed a validation detector combined with a convolutional neural network (CNN) for automatically segmenting document elements. Comparative trials against the Haar+Adaboost detector further highlighted the performance benefits of the proposed framework. Reference [4] introduced a three-stage seal validation scheme utilizing the StaVer dataset. Initially, the target field was segmented. Subsequently, this area was classified as seal or non-seal through a support vector machine (SVM), attaining an accuracy of 90%. Finally, the seal’s authenticity was further verified. Likewise, [30] investigated multiple YOLOv8 variants, particularly YOLOv8n, YOLOv8s, and YOLOv8m, to develop a seal detector that integrates high efficiency with precision. To evaluate model efficacy, the investigators utilized precision, recall, and mAP50 as performance metrics. The experimental findings demonstrated that YOLOv8n provided the most robust detection results, achieving an mAP of 98.63%.

During invoice creation, seal application, scanning, and storage, seal regions are often affected by occlusion, overlap, blur, discoloration, and illumination variations, substantially complicating reliable detection. To improve detection accuracy and meet industry requirements, researchers have developed numerous machine-learning and deep-learning methods for invoice seal localization. For example, [25] proposed a neural network-based framework for automatically identifying and classifying passport seals. In [26], a fully convolutional neural network was used to segment images into seal and non-seal regions. Built on a pretrained VGG-16 model and evaluated on the StaVer dataset, the method achieved 87% precision and 84% recall. However, the authors noted that the method continued to have difficulty segmenting black seals. Reference [27] developed YOLO-Stamp, a compact neural network architecture derived from the YOLO detector. With only 128,000 parameters, YOLO-Stamp achieved 90.6% accuracy and 90.7% recall on the scanned pseudo-official dataset (SPODS). Reference [28] proposed a generative adversarial network (GAN)-based process for extracting texture features from images containing seals. Building on this approach, an improved seal text recognition method, PP-OCR, was introduced to recognize text across different seal types. Similarly, [29] combined a validation detector with a convolutional neural network (CNN) to automatically segment document elements. Comparative experiments against the Haar+Adaboost detector demonstrated the performance advantages of the proposed framework. Reference [4] proposed a three-stage seal validation scheme using the StaVer dataset. The three-stage method first segmented the target region for subsequent classification. It then used a support vector machine (SVM) to classify the region as seal or non-seal, achieving an accuracy of 90%. In the final stage, the method further verified the authenticity of the detected seal. Reference [30] evaluated YOLOv8n, YOLOv8s, and YOLOv8m to develop an efficient and accurate seal detector. The investigators evaluated model performance using precision, recall, and mAP50. YOLOv8n achieved the strongest detection performance, with an mAP of 98.63%. Recent remote-sensing studies also provide complementary methodological insights for invoice seal detection. Hong et al. [31] modeled spatial-temporal proximity and topological relationships to distinguish densely distributed targets, offering a methodological reference for context-aware regional routing in C2BRA. Hong et al. [32] separated foreground and background motion features, informing the gated feature-selection mechanism in C3k2_CFC for suppressing table-line and text interference. Zhu et al. [33] combined geometry-aware enhancement with deformable sampling, providing a methodological reference for DAttention to capture boundary variations in circular and square seals. Han et al. [34] reviewed UAV vision applications and emphasized the need to jointly consider accuracy, efficiency, and deployment, which aligns with the design objectives of ISD-YOLO.

From the perspective of multi-scale feature fusion, lightweight YOLO-series detectors [10–13,20–24] generally employ FPN- or PAN-style necks. FPN transfers high-level semantic information to lower feature levels through a top-down pathway, but its unidirectional structure provides limited feedback of fine localization information to deeper layers. PAN introduces an additional bottom-up pathway and thereby improves bidirectional information exchange. However, its cross-scale connections are still constructed using predetermined upsampling, downsampling, and concatenation operations, without explicitly selecting informative spatial regions according to image content. BiFPN, as adopted in EfficientDet [14], further introduces learnable fusion weights, but these weights mainly operate at the feature-level scale and are generally shared across spatial locations. Transformer-based detectors, such as RT-DETR [35], establish long-range and cross-scale dependencies through attention mechanisms, but dense token interactions and complex encoding operations may increase the computational burden for lightweight edge deployment. Consequently, existing strategies still face a trade-off among multi-scale interaction, content-adaptive feature selection, and computational efficiency.

While current research has achieved significant advancements in seal identification, invoice stamp detection remains burdened by numerous obstacles. Within real-world scenarios, invoices usually undergo several phases, involving creation, sealing, digitization, and preservation, and are frequently impacted by various elements, including occlusion, overlapping, blurring, discoloration, and lighting shifts. Therefore, seal areas display intricate and fluctuating visual attributes, which greatly intensify the complexity of precise localization. Furthermore, invoice stamps are frequently interwoven with typography, tabular borders, and other document components. Additionally, certain seals show hazy boundaries, inconsistent tone patterns, and localized deterioration, additionally complicating the task for networks to pinpoint and identify stamp zones efficiently. Consequently, enhancing the classification precision and resilience of frameworks amidst the integrated impacts of intricate environments, degraded visual data, and diverse types of distortions continues to represent a major challenge in the invoice stamp verification field.

While current research has achieved significant advancements in seal identification, invoice seal detection remains burdened by numerous obstacles. Within real-world scenarios, invoices usually undergo several phases, involving creation, sealing, digitization, and preservation, and are frequently impacted by various elements, including occlusion, overlapping, blurring, discoloration, and lighting shifts. Therefore, seal areas display intricate and fluctuating visual attributes, which greatly intensify the complexity of precise localization. Furthermore, invoice seals are frequently interwoven with typography, tabular borders, and other document components. Additionally, certain seals show hazy boundaries, inconsistent tone patterns, and localized deterioration, additionally complicating the task for networks to pinpoint and identify seal regions efficiently. Consequently, enhancing the classification precision and resilience of frameworks amidst the integrated impacts of intricate environments, degraded visual data, and diverse types of distortions continues to represent a major challenge in the invoice seal verification field.

Against this background, to address insufficient multi-scale feature representation, severe interference from complex invoice backgrounds, and the difficulty of balancing detection accuracy with computational efficiency for potential edge deployment, this study proposes ISD-YOLO, a lightweight framework for invoice seal detection. ISD-YOLO adopts an efficient bidirectional feature-fusion topology and enhances the deep features entering this fusion pathway through content-adaptive regional routing and deformable sampling. Compared with unidirectional feature transfer or purely fixed cross-scale fusion, this design enables high-level semantic context and shallow localization details to complement each other in both directions, while reducing the propagation of irrelevant responses from invoice text, table lines, and local occlusions. Consequently, the proposed framework achieves a more effective balance among multi-scale representation, interference suppression, and computational efficiency. The main contributions of this study are summarized as follows:

1. To address the inadequate representation of multi-scale features in invoice seals, including overall structure, edge details, and internal text, the C3k2-ConvFormer-CGLU module (C3k2_CFC) is proposed. By integrating ConvFormer’s local feature enhancement capability with CGLU’s gated feature selection mechanism, the module adaptively enhances the local textures and global contours of invoice seals, thereby improving the model’s representation of fine-grained features.
2. To address large variations in seal scale, loss of edge details, and feature confusion caused by overlap with background text and table lines, a C2BRA module based on a bi-level routing attention mechanism was designed. By dynamically selecting key feature regions through coarse-grained and fine-grained routing strategies, the module enhances the perception of seal shape, edges, and internal textures while reducing computational redundancy. On this basis, a deformable attention module, DAttention, was introduced to further improve the model’s focus on key regions and effectively suppress interference from complex backgrounds.
3. To balance detection accuracy and computational efficiency in seal detection, we designed a lightweight detection head, EfficientHead. The module adopts a dual-branch parallel architecture and incorporates grouped convolution to efficiently extract multi-scale seal features, thereby jointly optimizing classification and localization while reducing the number of parameters and computational overhead. In addition, Distribution Focal Loss (DFL) is introduced to enhance seal-boundary localization under complex backgrounds and partial occlusion. By reducing model complexity while maintaining detection performance, this design offers potential for application in resource-constrained environments and for edge deployment.

The subsequent sections of this paper are organized in the following manner. Section 2 describes the integrated technical framework along with the proposed methodology. Section 3 presents and interprets the empirical results. Section 4 concludes the research.

## 2. Materials and methods

**Fig 1:**
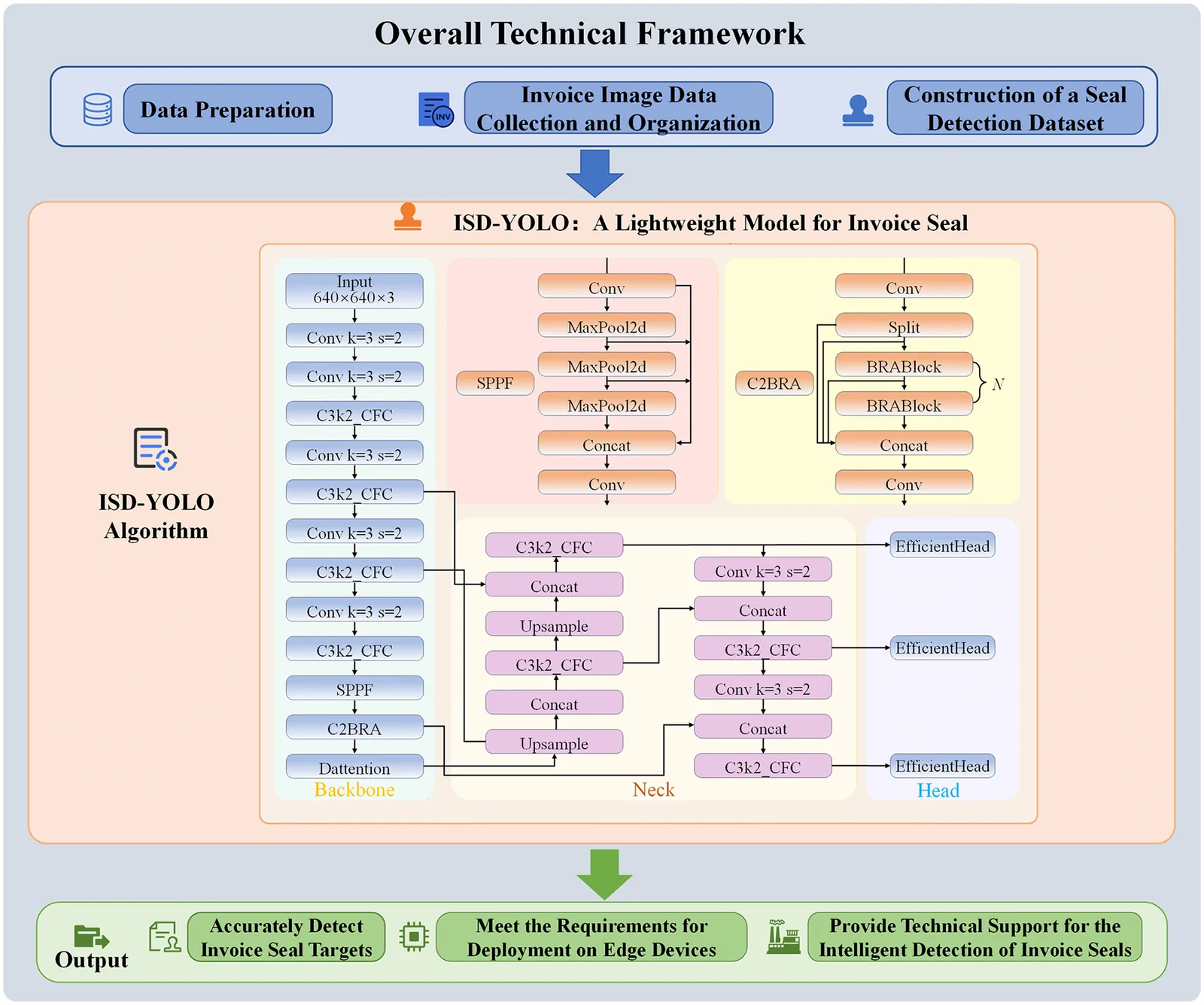
Overall technical framework.

This study focuses on invoice seal localization and introduces a compact detection architecture that balances detection accuracy and computational efficiency, with potential for future edge deployment. Fig 1 demonstrates the comprehensive technical architecture of the suggested approach. Initially, invoice image materials are gathered and structured to build a specialized seal identification dataset. The proposed ISD-YOLO framework is subsequently utilized for identifying and classifying seals within invoice photographs. Within this architecture, a C3k2_CFC unit is formulated to augment the backbone architecture’s fine-grained depiction of multi-level seal attributes, including global layouts and marginal details. By fusing ConvFormer’s localized feature intensification capacity with CGLU’s gated selection strategy, the unit mitigates inadequate feature manifestation under intricate backgrounds. Furthermore, a C2BRA unit grounded in a dual-layer routing attention scheme is integrated into the deeper levels of the backbone architecture. Utilizing coarse-grained and fine-grained two-phase routing tactics to selectively identify vital feature zones, the unit diminishes processing overhead while strengthening the network’s recognition of seal contours, boundaries, and internal patterns. Building upon this premise, the flexible attention module DAttention is further incorporated to hone the network’s emphasis on critical regions and effectively reduce interference from the backdrop. Ultimately, to balance inference efficiency and recognition precision while reducing computational requirements, a streamlined detection front-end, EfficientHead, is engineered. This component utilizes a dual-branch parallel layout and incorporates grouped convolutions to swiftly extract multi-scale seal characteristics, thus permitting cooperative refinement of categorization and localization while decreasing parameter volume and operational expenditure. In essence, the suggested technique provides an effective and lightweight solution for precise invoice seal recognition, and its reduced model complexity indicates potential for deployment on resource-constrained edge hardware. Its actual performance on edge devices, however, remains to be verified through dedicated hardware-level deployment experiments.

### 2.1 The proposed method

**Fig 2:**
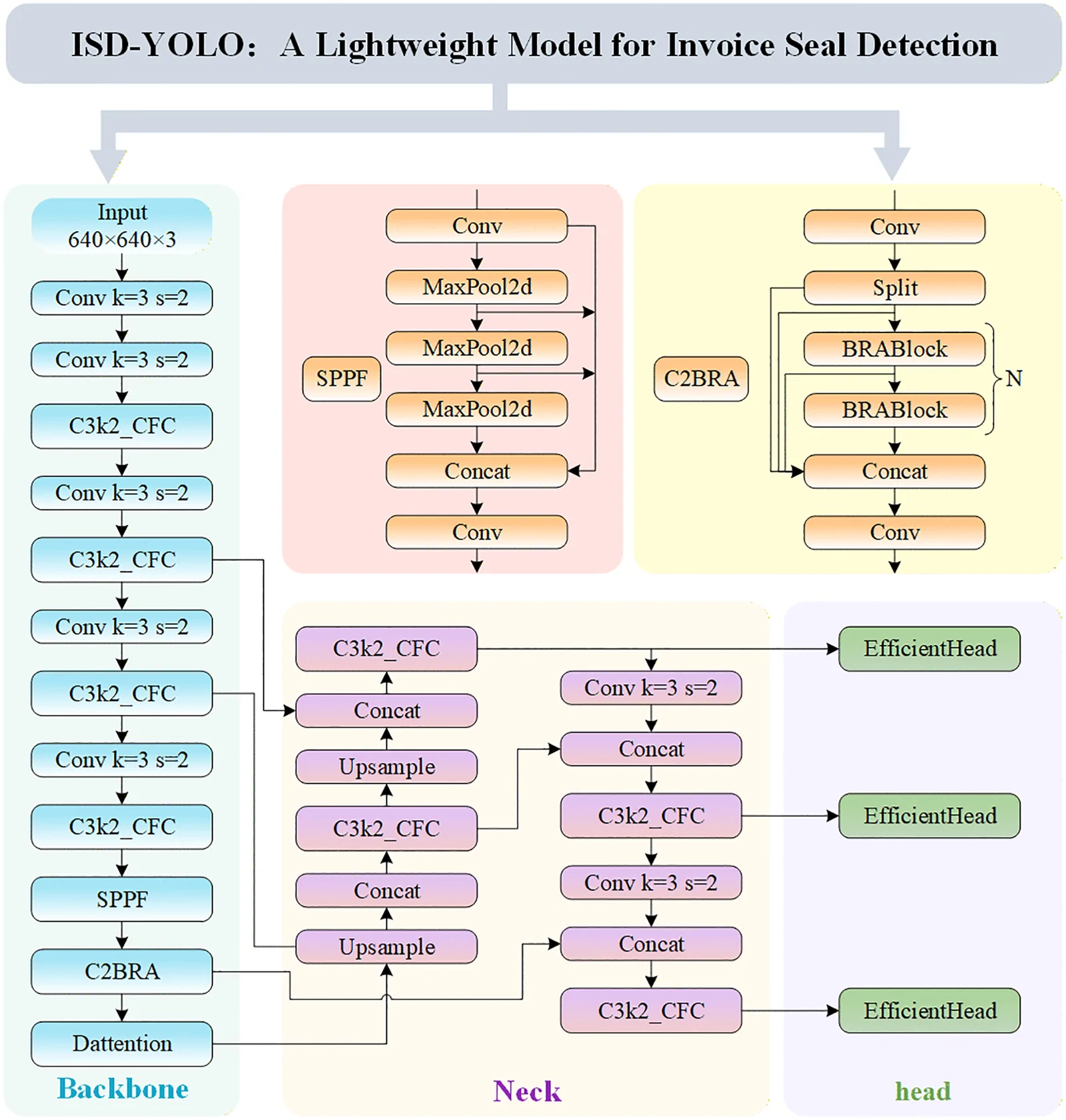
Network structure diagram of the ISD-YOLO model.

To address the characteristics of automated invoice seal detection, this paper proposes an efficient seal detection network, ISD-YOLO, as shown in Fig 2. The network is mainly designed from four perspectives: feature enhancement, attention optimization, global context modeling, and detection head lightweighting. In the backbone network, the standard building block in the C3k2 module is replaced with C3k2_CFC to improve the fine-grained representation of multi-scale seal features, such as global structure, edge details, and internal text. This module integrates ConvFormer’s local feature enhancement capability with the Convolutional Gated Linear Unit (CGLU) and adaptively enhances local seal textures and global contours through position-aware feature extraction and gated feature selection. Within the lower-tier backbone architecture, a C2BRA component utilizing Bi-level Routing Attention (BRA) is integrated to substitute the original C2PSA framework. Through coarse-grained and fine-grained two-stage routing strategies, this module dynamically selects key feature regions, enhances the perception of seal shape, edges, and internal textures, and reduces computational redundancy. Subsequently, a DAttention is introduced. Using a data-dependent dynamic sampling strategy, the model is further guided to focus on key seal regions, thereby effectively suppressing interference from background text and table lines. In the neck of the feature pyramid, C3k2_CFC is further deployed after the upsampling and concatenation operations to reorganize and enhance multi-scale features. In the detection head, a lightweight EfficientHead module is designed to reduce computational burden and support potential edge deployment. This framework utilizes a two-branch parallel structure, wherein a grouped convolutional stem is employed to capture multi-scale seal characteristics, whereas the localization and categorization branches handle detection and identification tasks independently. The localization module integrates Distribution Focal Loss, which represents bounding box coordinates as discrete probability distributions, thereby improving the localization accuracy of seal outlines that intersect with text or are partially obscured. Through the synergistic impact of these components, the model attains a robust equilibrium between architectural complexity and processing efficiency while maintaining the multi-scale feature extraction capability.

### 2.2 C3k2-ConvFormer-CGLU module

In automated invoice seal detection, the model must simultaneously capture multi-scale visual features. Specifically, it must perceive the global contour of the seal while distinguishing subtle boundary discontinuities, overlap with background text, and the distribution of internal seal text at the local level. To address this challenge efficiently, this section proposes a C3k2-ConvFormer-CGLU module, denoted as C3k2_CFC. By integrating ConvFormer’s global contextual modeling capability with CGLU’s gated feature selection mechanism, the proposed module enhances the network’s ability to discriminate multi-scale seal features.

The C3k2_CFC module is developed based on the C3k2 structure. Specifically, given an input feature map $X\in {\mathbb{R}}^{C\times H\times W}$, a standard convolution is first applied for preliminary feature extraction:


$$\begin{array}{c} X^{\prime }=Conv\left(X\right) \end{array}$$ (1)

Subsequently, $X^{\prime }$ is split along the channel dimension into N sub-feature maps:


$$\begin{array}{c} \left\{{X^{\prime }}_{\text{1}},\text{\textbackslash{}hspace\{0.33em}\}{X^{\prime }}_{\text{2}},\text{\textbackslash{}hspace\{0.33em}\ldots \},\text{\textbackslash{}hspace\{0.33em}\}{X^{\prime }}_{N}\right\}=Split_{ch}\left(X^{\prime }\right) \end{array}$$ (2)

Each sub-feature map is sequentially processed by multiple ConvFormerCGLUBlocks. Let $Z_{i}^{\left(0\right)}={X^{\prime }}_{i}$ denote the input of the *i-th* branch. When m ConvFormerCGLUBlocks are used, the recursive transformation is expressed as


$$\begin{array}{c} Z_{i}^{\left(t\right)}=CGLU\left(ConvFormer\left(Z_{i}^{\left(t-1\right)}\right)\right),\text{\textbackslash{}hspace\{0.33em}\}t=1,\text{\textbackslash{}hspace\{0.33em}\ldots \},\text{\textbackslash{}hspace\{0.33em}\}m, \end{array}$$ (3)

and the output of this branch is ${X^{\prime \prime \prime }}_{i}=Z_{i}^{\left(m\right)}$. The transformed sub-feature maps are then concatenated along the channel dimension and fused using a 1×1 convolution:


$$\begin{array}{c} Y=Conv\left(Concat_{ch}\left({X^{\prime \prime \prime }}_{\text{1}},\text{\textbackslash{}hspace\{0.33em}\}{X^{\prime \prime \prime }}_{\text{2}},\text{\textbackslash{}hspace\{0.33em}\ldots \},\text{\textbackslash{}hspace\{0.33em}\}{X^{\prime \prime \prime }}_{N}\right)\right) \end{array}$$ (4)

Through this design, the C3k2_CFC module preserves the efficient feature reorganization capability of C3k2 while progressively refining each branch through ConvFormer and CGLU. The module was incorporated into the backbone and neck of the baseline YOLO11n model to generate high-quality multi-scale features for the detection head.

(1) ConvFormer Module

**Fig 3:**
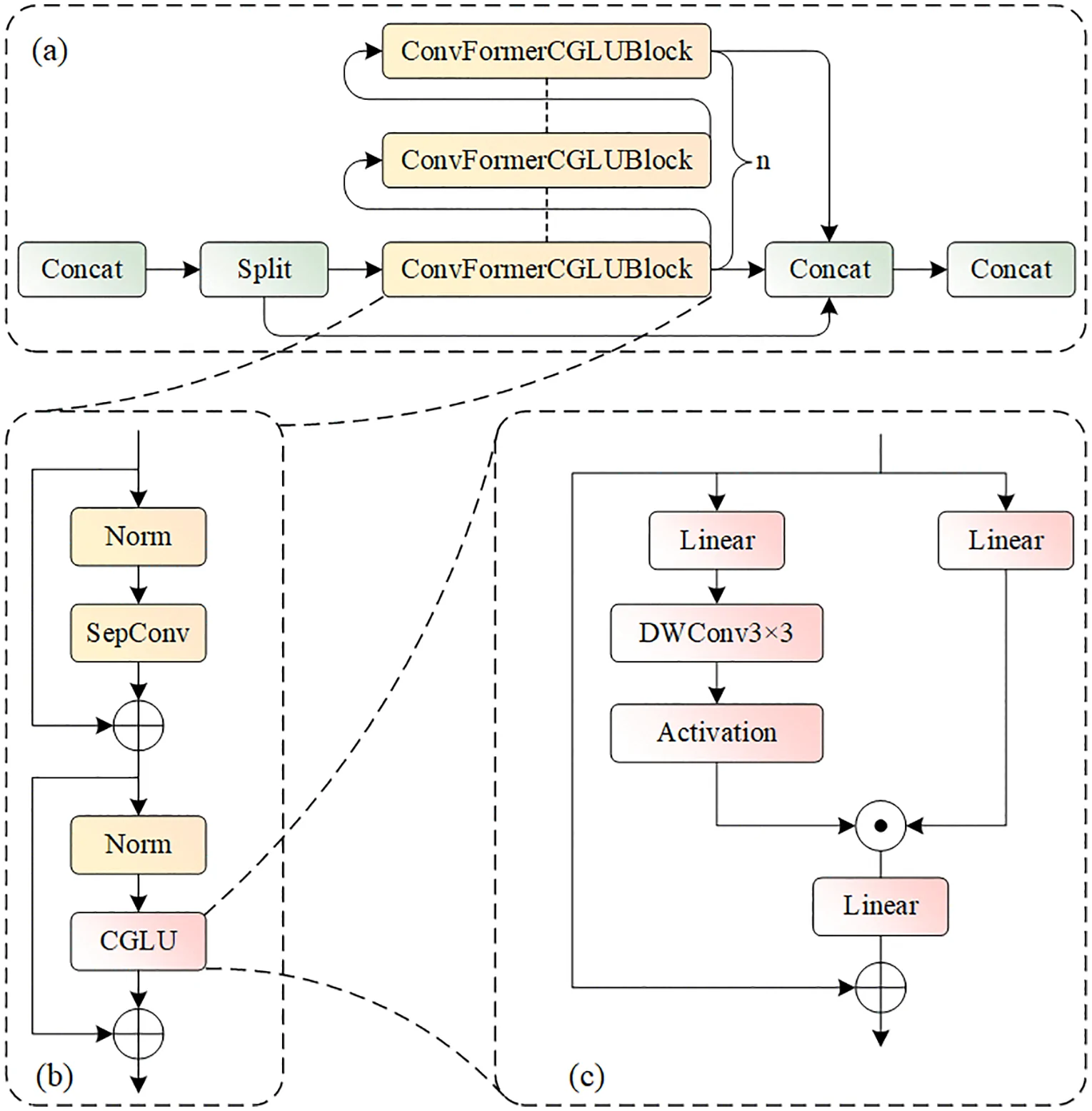
Block diagram of the ConvFormer module.


The ConvFormer module [36] is used to encode local positional information and aggregate contextual features, and its structure is illustrated in Fig 3b. Given the input feature $Z_{i}^{\left(t-1\right)}$, ConvFormer first stabilizes the feature distribution using LayerNorm and then employs Separable Convolution (SepConv) to capture local positional information. Its computational process is formulated as:

$$\begin{array}{c} {\tilde{Z}}_{i}^{\left(t\right)}=Z_{i}^{\left(t-1\right)}+SepConv\left(LayerNorm\left(Z_{i}^{\left(t-1\right)}\right)\right) \end{array}$$ (5)

SepConv combines depthwise and pointwise convolutions:


$$\begin{array}{c} SepConv\left(A\right)=PWConv_{1\times 1}\left(DWConv_{k_{S}\times k_{S}}\left(A\right)\right) \end{array}$$ (6)

The depthwise convolution captures spatial information independently within each channel, whereas the pointwise convolution performs cross-channel fusion. Compared with standard convolution, this structure reduces computational cost while retaining local feature extraction capability. The residual connection preserves the original feature representation and prevents weak seal-edge responses from being completely replaced by the transformed features.

(2) CGLU Module

The Convolutional Gated Linear Unit (CGLU) performs gated nonlinear transformation and feature selection on the features generated by ConvFormer [37]. CGLU integrates the advantages of Gated Linear Units with convolutional operations and introduces neighborhood-dependent channel modulation. Unlike the SE mechanism, which generates a spatially shared channel-weight vector through global average pooling, CGLU derives a different gating response for each spatial position from its local neighboring features.

Given the ConvFormer output ${\tilde{Z}}_{i}^{\left(t\right)}$, the content and gating branches are defined as:


$$\begin{array}{c} A_{i}^{\left(t\right)}={\tilde{Z}}_{i}^{\left(t\right)}W_{\text{1}}+B_{\text{1}},G_{i}^{\left(t\right)}=GELU\left(DWConv_{3\times 3}\left({\tilde{Z}}_{i}^{\left(t\right)}W_{\text{2}}+B_{\text{2}}\right)\right) \end{array}$$ (7)

The CGLU output is then obtained through element-wise multiplication:


$$\begin{array}{c} Z_{i}^{\left(t\right)}=A_{i}^{\left(t\right)}⊙G_{i}^{\left(t\right)} \end{array}$$ (8)

Here, $W_{\text{1}}$ and $W_{\text{2}}$ denote the linear projection matrices, $B_{\text{1}}$ and $B_{\text{2}}$ denote the bias terms, $DWConv_{3\times 3}(\cdot )$ represents a 3×3 depthwise convolution, GELU(·) denotes the Gaussian Error Linear Unit, and ⊙ represents element-wise multiplication. In practice, the bias terms can be omitted to reduce computational complexity.

The multi-scale representation capability of C3k2_CFC can be explained by the different receptive fields generated by the sequential blocks and feature-pyramid levels. Let $R_{l,0}$ denote the initial receptive-field diameter at pyramid level $l,\text{\textbackslash{}hspace\{0.33em}\}s_{l}$ denote the corresponding feature stride, and $k_{s}$ denote the spatial kernel size. After t local transformations, the approximate receptive field is:


$$\begin{array}{c} R_{l,t}=R_{l,0}+t(k_{s}-1)s_{l} \end{array}$$ (9)

Therefore, shallow feature levels retain fine boundary and internal-text information for small seals, whereas deeper levels provide a larger contextual field for representing the overall contours of large seals. The final concatenation and 1×1 fusion operation preserve features with different effective receptive fields rather than retaining only the deepest representation.

For partially occluded seals, the local gating response $G_{i}^{\left(t\right)}$ is generated from neighboring features. Consequently, text, table lines, or other responses that are inconsistent with the surrounding seal structure can be attenuated during element-wise modulation, while visible seal contours and internal patterns can be retained or enhanced. Combined with the residual feature propagation in ConvFormer and the partial feature path of C3k2, this mechanism improves the model’s tolerance to partial occlusion. However, it does not imply that the module can reconstruct seal information that is completely absent from the input image.

### 2.3 C2BRA module

Within YOLO11, the C2PSA framework plays an important role in feature extraction by integrating the Partial Self-Attention (PSA) substructure. This mechanism employs a Cross Stage Partial (CSP) architecture to divide the feature map into two branches: one branch recalibrates the features through PSA, whereas the other preserves the projected input features, after which the outputs of the two branches are combined. Nevertheless, although PSA effectively captures contextual information, channel compression in its attention branch may weaken fine-grained details of small-scale targets. In invoice seal detection, seal sizes vary considerably and the seal regions frequently overlap with text and table lines; therefore, such information loss may reduce detection accuracy.

**Fig 4:**
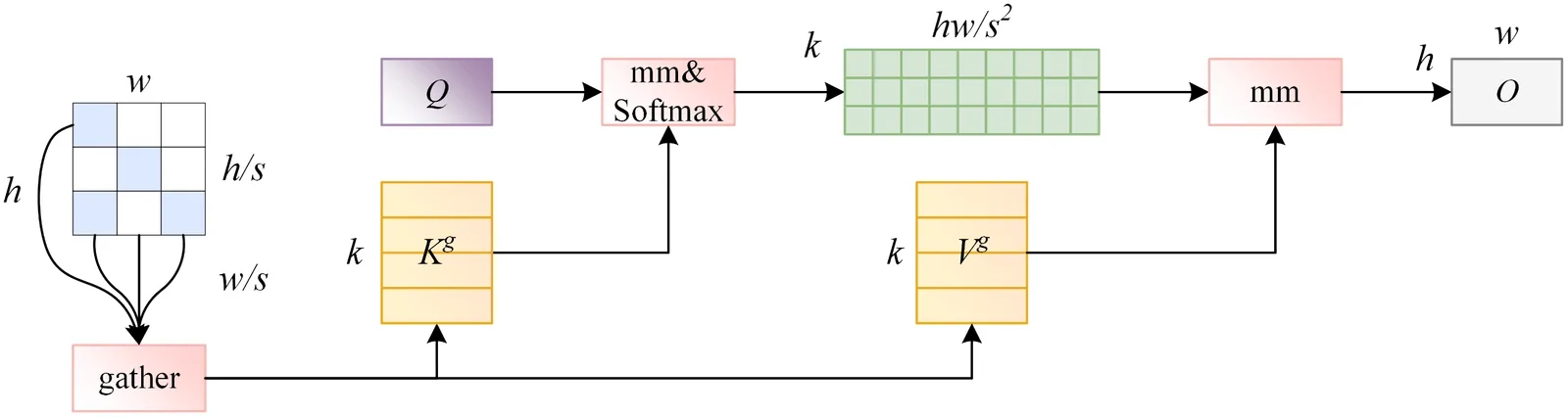
Block diagram of the BRA mechanism.


To address this problem, this study integrates Bi-Level Routing Attention (BRA) into YOLO11 to construct the C2BRA module. Proposed by Zhu et al. [38], BRA performs efficient attention computation through coarse-grained region routing followed by fine-grained token attention, as illustrated in Fig 4. For the input $Z\in {\mathbb{R}}^{h\times w\times c}$ of a BRA block, the spatial dimensions are first flattened, and the query, key, and value features are obtained through linear projections:

$$\begin{array}{c} Q=Z_{f}W_{Q},\text{\textbackslash{}hspace\{0.33em}\}K=Z_{f}W_{K},V=Z_{f}W_{V},Q,K\in {\mathbb{R}}^{hw\times d_{v}},\text{\textbackslash{}hspace\{0.33em}\}V={\mathbb{R}}^{hw\times d_{v}} \end{array}$$ (10)

Where The feature map $Z_{f}=Flatten\left(Z\right)$ is then divided into $R=hw/s^{\text{2}}$ non-overlapping regions, each containing $M=s^{\text{2}}$ tokens. For query region r and candidate region j, average pooling is used to obtain region-level descriptors. Their similarity and the corresponding Top-*k* routing indices are calculated as:


$$\begin{array}{c} {\overset{―}{q}}_{r}=\frac{\text{1}}{M}\sum_{p=1}^{M}Q_{r,p},\text{\textbackslash{}hspace\{0.33em}{\overset{―}{k}}_{j}=\frac{\text{1}}{M}\}\sum_{p=1}^{M}K_{j,p},S_{r,j}=\frac{{\overset{―}{q}}_{r}{\overset{―}{k}}_{j}^{T}}{\sqrt{d_{k}}},J_{r}=Top\text{\textbackslash{}hspace\{0.33em}\}K_{j}\left(S_{r,j},k\right) \end{array}$$ (11)

The indices $J_{r}$ identify the *k* regions most relevant to query region *r*. The token-level keys and values in these regions are subsequently gathered without spatial compression:


$$\begin{array}{c} K_{r}^{g}=Concat_{j\in J_{r}}\left(K_{j}\right),V_{r}^{g}=Concat_{j\in J_{r}}\left(V_{j}\right) \end{array}$$ (12)

Where $K_{r}^{g}\in {\mathbb{R}}^{kM\times d_{k}}$ and $V_{r}^{g}\in {\mathbb{R}}^{kM\times d_{k}}$ Fine-grained attention is then computed only between the query tokens in region r and the tokens gathered from its routed regions:


$$\begin{array}{c} A_{r}=Softmax\left(\frac{Q_{r}(K_{r}^{g}{)}^{T}}{\sqrt{d_{k}}}\right) \end{array}$$ (13)

$$\begin{array}{c} O_{r}=A_{r}V_{r}^{g},\text{\textbackslash{}hspace\{0.33em}\}O=Fold\left(O_{\text{1}},\text{\textbackslash{}hspace\{0.33em}\}O_{\text{2}},\text{\textbackslash{}hspace\{0.33em}\ldots \},\text{\textbackslash{}hspace\{0.33em}\}O_{R}\right) \end{array}$$ (14)

Here, $d_{k}$ denotes the dimensionality of the key vectors, and division by $\sqrt{d_{k}}$ prevents excessively large dot products and stabilizes training. Compared with standard self-attention, whose attention complexity is $O((hw{)}^{\text{2}}d_{k})$, BRA requires:


$$\begin{array}{c} O(R^{\text{2}}d_{k}+kRM^{\text{2}}d_{k})=O\left(\frac{(hw{)}^{\text{2}}}{s^{\text{4}}}d_{k}+k\left(hw\right)s^{\text{2}}d_{k}\right) \end{array}$$ (15)

Under the balanced routing setting $s^{\text{2}}=O((hw{)}^{1/3})$, with k and $d_{k}$ treated as constants, this complexity becomes $O((hw{)}^{4/3})$ Thus, BRA reduces redundant token interactions by restricting fine-grained attention to the routed regions while retaining token-level details.

**Fig 5:**
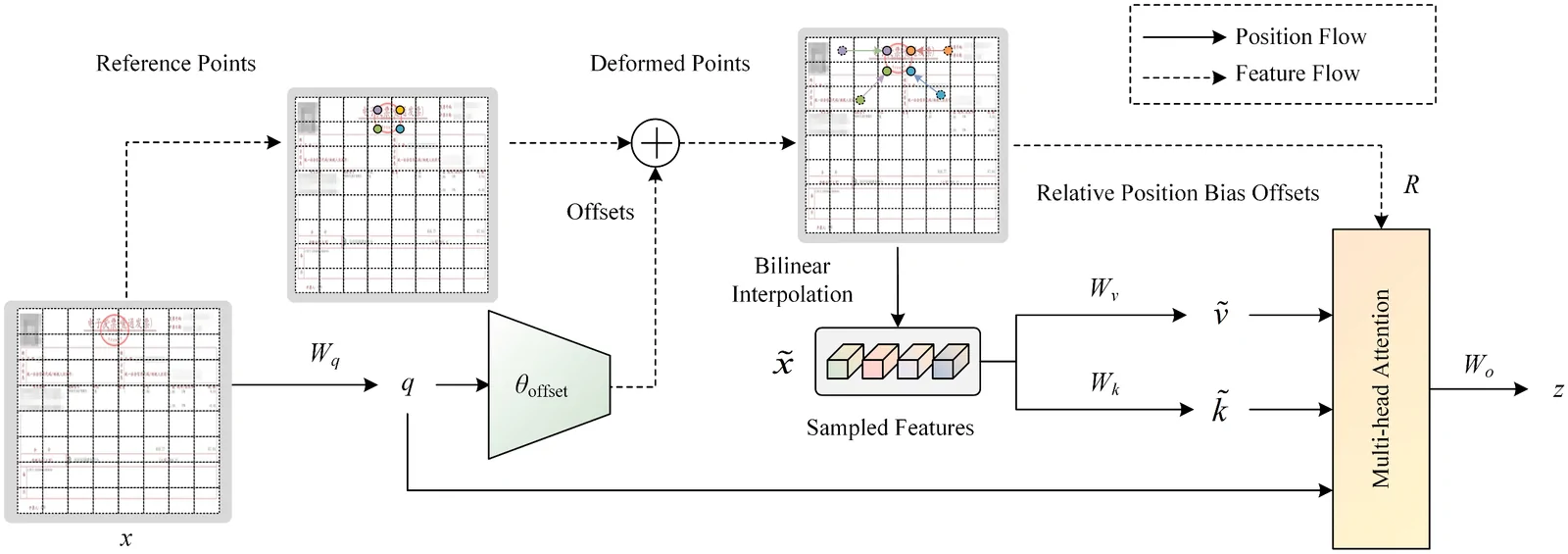
Block diagram of the C2BRA module.


The C2BRA module retains the CSP structure of C2PSA but replaces its internal PSA blocks with BRA blocks. As shown in Fig 5, its complete forward process can be expressed as

$$\begin{array}{c} \begin{array}{c} U=Conv_{1\times 1}^{in}\left(X\right),\left[U_{a},U_{b}\right]=Split_{ch}\left(U\right)\\ B^{\left(0\right)}=U_{b},B^{\left(t\right)}=BRABlock_{t}\left(B^{\left(t-1\right)}\right),t=1,\text{\textbackslash{}ldots},N\\ Y=Conv_{1\times 1}^{out}\left(Concat_{ch}\left(U_{a},B^{\left(N\right)}\right)\right) \end{array} \end{array}$$ (16)

The bypass branch $U_{a}$ preserves the projected input features, whereas $U_{b}$ is adaptively recalibrated by the BRA blocks. Their concatenation and subsequent 1×1 convolution combine the original local evidence with sparsely aggregated contextual information.

For invoice seal detection, the two attention levels provide complementary representations for targets of different scales. Fine-grained token attention preserves seal boundaries, strokes, and internal-text features that are important for small seals, whereas coarse region routing connects spatially separated contour regions and captures the overall structure of large seals. When C2BRA is deployed on deeper feature maps, each region corresponds to a larger area of the input image, thereby strengthening global shape representation and complementing the shallow features used in the feature pyramid. Moreover, background text and table-line regions outside the selected Top-*k* routes do not participate in the fine-grained attention calculation for the current query. For a partially occluded seal, visible contour and texture fragments can therefore aggregate complementary information from other relevant regions, while the CSP bypass retains local evidence that may be weakened by attention recalibration. Consequently, C2BRA can reduce interference from complex backgrounds and improve tolerance to partial occlusion, although it does not explicitly reconstruct completely missing seal information.

### 2.4 Deformable attention module

To improve the detection accuracy of the baseline YOLO11n framework, we integrate the Deformable Attention Module (DAttention) [39] into its feature-extraction backbone. Although conventional self-attention mechanisms can capture long-range dependencies, they incur substantial computational overhead and can be distracted by irrelevant background features. By contrast, conventional convolution uses a fixed receptive field and therefore struggles to capture both the global shape characteristics and fine-grained texture details of seals. DAttention dynamically adjusts sampling locations in a data-driven manner, enabling the model to focus on seal-relevant regions while suppressing background clutter in invoices.

Given an input feature map $x\in {\mathbb{R}}^{H\times W\times C}$, a uniformly distributed reference grid $p\in {\mathbb{R}}^{H_{G}\times W_{G}\times 2}$ is first generated, where $H_{G}=H/r,\text{\textbackslash{}hspace\{0.33em}\}W_{G}=W/r$ and $N_{s}=H_{G}W_{G}$. The reference coordinates are normalized to [−1,1]. The query feature is obtained as $q=xW_{q}$, where $W_{q}$ denotes the query projection matrix. A lightweight offset network, consisting of a 5×5 depthwise convolution followed by a 1×1 convolution, predicts a two-dimensional displacement for each reference point:


$$\begin{array}{c} \Delta p=stanh\left(\theta_{offset}\left(q\right)\right),\hat{p}=p+\Delta p \end{array}$$ (17)

Here, s controls the maximum displacement, and $\hat{p}$ denotes the deformed sampling locations. Because Δp is generated from the query features, both the direction and magnitude of the sampling displacement vary according to the local seal content.

The sampled feature and its corresponding key and value projections are then obtained by


$$\begin{array}{c} \tilde{x}=\phi \left(x,\hat{p}\right),\tilde{k}=\tilde{x}W_{k},\tilde{v}=\tilde{x}W_{v} \end{array}$$ (18)

Where ϕ(·,·) denotes bilinear interpolation. Before interpolation, a normalized sampling coordinate $\left(a_{x},a_{y}\right)$ is mapped to feature-map coordinates as $((W-1)\left(a_{x}+1)/2,(H-1\right)(a_{y}+1)/2)$. For the sampling point ${\hat{p}}_{j}=\left({\hat{p}}_{j,x},{\hat{p}}_{j,y}\right)$, bilinear interpolation is calculated from its four neighboring integer positions $N\left({\hat{p}}_{j}\right)$:


$$\begin{array}{c} \phi (x;{\hat{p}}_{j})=\sum_{\left(r_{x},r_{y}\right)\in N\left({\hat{p}}_{j}\right),y}g\left({\hat{p}}_{j,x},r_{x}\right)g\left({\hat{p}}_{j,y},r_{y}\right)x[r_{y},r_{x},:] \end{array}$$ (19)

$$\begin{array}{c} g\left(a,b\right)=max(0,\text{\textbackslash{}hspace\{0.33em}\}1-|a-b\left|\right) \end{array}$$ (20)

The bilinear interpolation weights are evaluated at the deformed sampling positions. Because the interpolation kernel is piecewise differentiable, gradients from the detection loss can propagate through the sampled features to the predicted offsets and the offset network. Consequently, during training, the offset predictor learns to shift the sampling locations toward informative seal contours and visible texture regions.

After flattening the spatial dimensions, $q\in {\mathbb{R}}^{H\times W\times C}$ and $\tilde{k},\tilde{v}\in {\mathbb{R}}^{N_{s}\times C}$. Let M denote the number of attention heads and d=C/M the dimensionality of each head. For the m-th head, the attention logits, normalized weights, and output are calculated as


$$\begin{array}{c} \begin{array}{c} e_{i,j}^{\left(m\right)}=\frac{q_{i}^{\left(m\right)}{\tilde{k}}_{j}^{\left(m\right)T}}{\sqrt{d}}+\phi ({\hat{B}}^{\left(m\right)};R_{i,j}),\\ \alpha_{i,j}^{\left(m\right)}=\frac{exp\left(e_{i,t}^{\left(m\right)}\right)}{\sum_{t=1}^{N_{s}}exp\left(e_{i,t}^{\left(m\right)}\right)},\\ z_{i}^{\left(m\right)}=\sum_{j=1}^{N_{s}}a_{i,j}^{\left(m\right)}{\tilde{v}}_{j}^{\left(m\right)}. \end{array} \end{array}$$ (21)

Here, $R_{i,j}$ denotes the continuous relative displacement between query position i and deformed sampling point j. The term $\phi ({\hat{B}}^{\left(m\right)};R_{i,j})$ is obtained by interpolating the learnable relative-position bias table ${\hat{B}}^{\left(m\right)}\in {\mathbb{R}}^{\left(2H-1)(2W-1\right)}$, enabling the positional bias to accommodate continuously changing sampling locations. Finally, the outputs of all heads are concatenated and projected to obtain:


$$\begin{array}{c} z=Reshape_{H\times W\times C}\left(Concat\left(z^{\left(1\right)},\text{\textbackslash{}ldots},z^{\left(M\right)}\right)W_{o}\right) \end{array}$$ (22)

Excluding the query and output projections shared with conventional attention, the computational complexity of deformable multi-head attention is:


$$\begin{array}{c} \Omega \left(DMHA\right)=2HWN_{s}C+2N_{s}C^{\text{2}}+({\hat{k}}^{\text{2}}+2)N_{s}C \end{array}$$ (23)

where $\hat{k}=5$ is the kernel size of the offset network and $N_{s}=H_{G}W_{G}=HW/r^{\text{2}}$. Substitution gives a dominant attention term of $2(HW{)}^{\text{2}}C/r^{\text{2}}$. Therefore, deformable attention reduces the dense attention cost by approximately a factor of $r^{\text{2}}$; it becomes linear in H×W only when $N_{s}$ is fixed independently of the input resolution. Its deployment on the low-resolution deep feature map further limits the practical computational cost.

**Fig 6:**
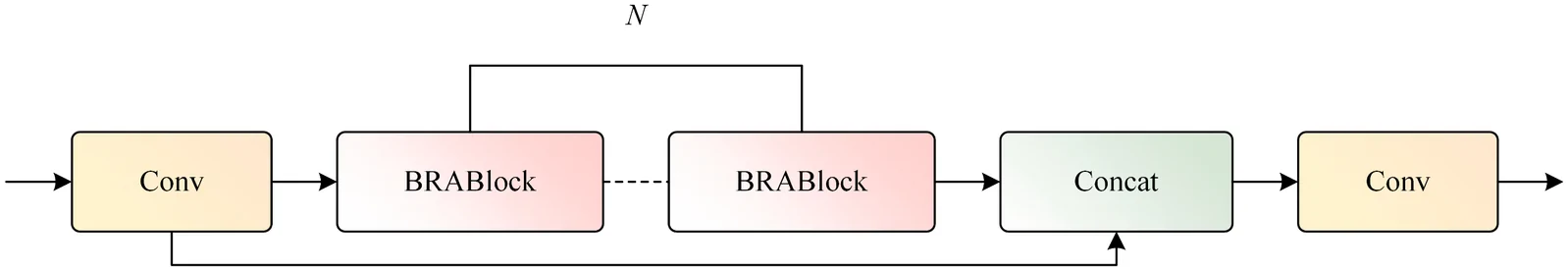
Architecture of the DAttention module.

The theoretical adaptability of the DAttention to seals at different scales stems from its content-dependent offsets. Small seal features can generate compact sampling patterns that preserve local boundaries, strokes, and internal text, whereas larger or rotated seals can generate directional displacements that connect spatially separated contour regions. In ISD-YOLO, the DAttention is placed after C2BRA on the deepest backbone feature. Its output is then propagated through the top-down neck and fused with the shallower and feature levels. Consequently, the global contextual information enhanced by the DAttention complements the high-resolution boundary details retained by the shallow feature levels. The architecture of the DAttention is shown in Fig 6.

For partially occluded seals, the differentiable bilinear sampling operation allows detection-loss gradients to optimize the offset predictor, encouraging the sampling points to shift toward visible contour and texture fragments. Meanwhile, background or occluding features tend to receive lower attention weights when their semantic similarity to the query is weak or their relative spatial arrangement is inconsistent with the seal geometry. The learnable relative-position bias further favors geometrically consistent feature aggregation. Consequently, DAttention can attenuate interference from invoice text, table lines, and local occluders while aggregating complementary visible seal evidence. However, it cannot reconstruct seal information that is completely invisible.

### 2.5 Efficient head

To reduce the computational cost of YOLO11 for invoice seal detection and improve its potential for deployment in resource-constrained environments, this study introduces a lightweight detection head, named EfficientHead. Traditional detection heads may introduce considerable computational overhead, which can limit their suitability for resource-constrained hardware such as document scanners or mobile devices. EfficientHead reconstructs the feature-extraction framework by incorporating a refined lightweight convolutional structure. This approach diminishes the total parameter count and operational load while maintaining the essential acquisition and integration of semantic information for diverse seal categories.

This module processes multi-scale seal feature maps from all levels of the feature pyramid simultaneously. At each scale, features are first passed through the stem submodule, which contains two consecutive 3×3 grouped convolutions. The number of groups is dynamically set to one-sixteenth of the input channels. The first grouped convolution significantly reduces computational cost while extracting local texture and edge information of the seals. The second grouped convolution further fuses these features, enhancing nonlinear representation and improving the model’s ability to distinguish seals from background text and table lines. Compared with standard convolutions, this design achieves higher computational efficiency while effectively capturing features of both small square and large circular seals. The features enhanced by the stem submodule are then sent simultaneously to the regression and classification branches. The regression branch predicts bounding box distribution parameters for each spatial location using 1×1 convolutional layers, achieving precise localization of seal regions. Meanwhile, the classification branch outputs seal category confidence for each anchor point via 1×1 convolutional layers, enabling multi-class seal recognition. This dual-branch design enables the model to perform high-precision localization and reliable classification simultaneously in a single forward pass. Bounding box parameters from the regression branch are further refined by the distribution focal loss module, which models positions as discrete probability distributions. This approach effectively improves localization accuracy for seal edges that are partially occluded or overlapping with text. In invoice images, seals often overlap with text, numbers, or company names, making precise boundary definition challenging for traditional regression methods. The distribution focal loss module significantly mitigates this issue through probabilistic modeling.

In the validation experiments on invoice seal detection, the model equipped with EfficientHead demonstrated clear advantages. First, this design effectively controls model complexity and improves inference efficiency, providing favorable computational characteristics for potential deployment on resource-constrained edge devices. Second, by selectively optimizing feature flow, the model maintains high sensitivity and discriminative capability for seals of multiple scales and diverse forms. It also exhibits strong robustness to seals with varying rotation angles or partial blur. These results indicate that EfficientHead achieves an effective balance between speed and accuracy in automated financial document processing and has potential for future edge deployment; however, this potential has not yet been validated through dedicated tests on actual edge hardware.

## 3. Experimental results

### 3.1 Evaluation metrics

To thoroughly assess the effectiveness of the ISD-YOLO framework regarding detection precision and computational efficiency, precision (P), recall (R), F1-score, average precision (AP), mean average precision (mAP), GFLOPs, and FPS were utilized as benchmarking indicators. Precisely, P and R were employed to gauge the reliability and exhaustiveness of the network in object localization, accordingly; the F1-score was utilized to holistically describe the equilibrium between P and R; AP served to appraise the identification capability of the framework for each class; mAP, which denotes the average AP across all classes, was adopted to demonstrate the general detection quality of the system; GFLOPs was utilized to evaluate the arithmetic complexity of the architecture; and FPS signifies the quantity of frames that the system can handle per second and was utilized to determine the processing velocity. The detailed formulations of these metrics are provided in Eqs. (24)-(28).


$$\begin{array}{c} P=\frac{TP}{TP+FP} \end{array}$$ (24)

$$\begin{array}{c} F1-score=2\cdot \frac{P\cdot R}{P+R} \end{array}$$ (25)

$$\begin{array}{c} AP=\underset{\text{0}}{\int^{\text{1}}}P\left(R\right)dR \end{array}$$ (26)

$$\begin{array}{c} mAP=\frac{\text{1}}{m}\sum_{i=1}^{m}AP_{i} \end{array}$$ (27)

$$\begin{array}{c} FPS=\frac{F_{n}}{T} \end{array}$$ (28)

In this scenario, TP denotes the amount of correctly classified positive examples, FP refers to the frequency of negative samples incorrectly categorized as positive, whereas FN represents the total of positive subjects incorrectly marked as negative. AP is obtained from the P-R graph plotted with R across the x-axis and P across the y-axis, and its value is defined as the area encompassed by the curve. $F_{n}$ denotes the number of images to be detected, and T denotes the total time required to complete the detection.

### 3.2 Datasets and model training

To construct a high-quality invoice-seal detection dataset, this study used two data sources: a public dataset and a self-constructed dataset. The public dataset was obtained from the Kaggle and Roboflow platforms (links: https://www.kaggle.com/datasets/rtatman/stamp-verification-staver-dataset and https://universe.roboflow.com/marcos-7aslt/stampdet/dataset/10) and covered scenarios such as complex backgrounds and partial occlusion, with a total of 1,096 images. Among them, 732 images with seal annotations were obtained from Roboflow, whereas the remaining 364 images were selected from Kaggle and manually annotated. The self-constructed dataset consisted of 500 invoice-seal images provided by a collaborating enterprise. To standardize the data format, these 500 images were resized to 640 × 640 pixels and saved in JPG format. The two datasets contained a total of 1,596 images and were divided into training, validation, and test sets at a ratio of 7:2:1.

On this basis, Table 1 further summarizes the numbers of images, seal instances, and bounding boxes; the original image resolutions; data sources; image-level sample attributes; and seal types for the two datasets. Both datasets were configured as single-class object-detection datasets, with “seal” used as the unified class label. Because each image contained at least one annotated seal, the datasets included 1,596 positive images, and no separate negative images were included. The datasets contained complex backgrounds involving text, graphic patterns, and tables, as well as blurred and partially occluded seals. In some real invoice images, the seals also overlapped with surrounding text or table structures. Because the datasets and annotation files did not contain separate labels for challenging conditions such as faint seals, blur, occlusion, rotation, text overlap, and complex backgrounds, these conditions are described qualitatively in this study, without reporting their specific numbers or proportions. In addition, the public dataset was not further categorized by invoice type, and the self-constructed dataset was not further subdivided by seal type. Regarding annotation, the 732 Roboflow images retained the original annotations provided with the dataset, whereas the 364 Kaggle images and 500 self-constructed invoice-seal images were manually annotated using LabelImg. The manually generated annotation files were initially saved in XML format and subsequently converted into YOLO-format TXT files. For partially occluded seals, bounding boxes were drawn around the visible and identifiable regions of the seals, without extrapolating the invisible portions behind the occlusions.

**Table 1:** Summary of the datasets.

Dataset and Source	Dataset Purpose	Number of Images	Number of Annotated Bounding Boxes	Image Resolution	Positive/Negative Samples	Seal Type
Public seal dataset from the Roboflow and Kaggle platforms	Model training and quantitative evaluation	1,096	1,740/1,740	640 × 640	1,096/0	seal
Self-built invoice seal dataset; provided by the cooperating enterprise	Model training and qualitative evaluation	500	575/575	640 × 640	500/0	seal
Total: two datasets	Model training, testing, and evaluation	1,596	2,315/2,315	640 × 640	1,596/0	seal

Regarding the experimental framework, all experiments were implemented using the Ultralytics source code and initialized with the COCO-pretrained yolo11n.pt weights. The software environment consisted of Python 3.13.5, PyTorch 2.8.0, and CUDA 12.9, and an NVIDIA GeForce RTX 5070 Ti GPU with 12 GB of memory was used for acceleration. SGD was adopted with an initial learning rate of 0.01, a final learning-rate factor of 0.01, momentum of 0.937, and weight decay of 0.0005. The models were trained for 200 epochs with a batch size of 32 and an input resolution of 640 × 640. The random seed was fixed at 0 with deterministic computation enabled. Data augmentation included HSV adjustment (0.015, 0.7, and 0.4), translation (0.1), scaling (0.5), horizontal flipping (0.5), and Mosaic augmentation (1.0), which was disabled during the final 10 epochs; the remaining geometric and mixed-sample augmentations were disabled. Quantitative evaluation was conducted on the fixed test set using a confidence threshold of 0.001, an NMS IoU threshold of 0.70, and a maximum of 300 detections per image. All comparison models were trained using the same data split and experimental protocol. The detailed settings are summarized in Table 2.

**Table 2:** Training hyperparameter settings.

Parameter	Value
optimizer	SGD
lr0	0.01
lrf	0.01
momentum	0.937
weight_decay	0.0005
image_size	640
epochs	200
batch	32

### 3.3 Ablation experiments

To quantify the individual and cumulative contributions of the four introduced modules, we conducted systematic ablation experiments. All experiments used the same software and hardware environment and training hyperparameters as those described in Section 3.2 to ensure comparability. To eliminate the influence of differing training conditions, all ablation experiments employed the complete training configuration specified in Section 3.2, without independently tuning the hyperparameters for different module combinations. This setup ensured fair comparisons across experimental groups, so that performance changes primarily reflected the contributions of the introduced modules. As shown in Table 3, C3k2_CFC, C2BRA, DAttention, and EfficientHead were first evaluated individually, followed by progressive evaluations of their combinations. The symbols “√” and “×” denote that the corresponding module was enabled and disabled, respectively. The baseline model achieved an mAP50 of 95.1% and an mAP50:95 of 77.5%, with 2.6 M parameters, a computational cost of 6.3 GFLOPs, and an inference speed of 133.4 FPS.

**Table 3:** Ablation experiments for each module in the proposed method.

C3k2_CFC	C2BRA	DAttention	EfficientHead	mAP50 (%)	mAP50:95(%)	Params(M)	GFLOPS	FPS
×	×	×	×	95.1	77.5	2.6	6.3	133.4
√	×	×	×	96.8	79.4	2.3	6.1	132.4
×	√	×	×	96.2	77.8	2.6	6.3	161.7
×	×	√	×	95.4	77.8	2.8	6.5	132.1
×	×	×	√	96.0	78.1	2.3	5.1	181.3
√	√	×	×	96.5	78.5	2.3	6.0	143.1
√	√	√	×	96.9	79.2	2.7	6.4	139.7
√	√	√	√	97.2	79.8	2.3	5.0	158.0

These modules address different optimization dimensions and are therefore complementary rather than redundant. C3K2-CFC primarily improves feature representation by combining local convolutional encoding with contextual feature interaction. C2BRA performs content-adaptive regional routing and selectively emphasizes informative regions, thereby suppressing irrelevant background responses without increasing the parameter count or GFLOPs. DAttention focuses on spatially adaptive feature sampling and is intended to improve localization when seal contours are incomplete or displaced. EfficientHead operates at the prediction head and reduces computational redundancy, thereby improving inference efficiency. Thus, the four modules enhance feature representation, regional selection, spatial alignment, and prediction efficiency, respectively.

When introduced individually, C3k2_CFC increased mAP50 and mAP50:95 by 1.7 and 1.9 percentage points, respectively, while reducing the parameter count from 2.6 M to 2.3 M and GFLOPs from 6.3 to 6.1. Its FPS decreased slightly to 132.4, indicating that the accuracy improvement was achieved with almost unchanged inference speed. C2BRA improved mAP50 from 95.1% to 96.2% and mAP50:95 from 77.5% to 77.8% without increasing the parameter count or GFLOPs, while FPS increased to 161.7. This result demonstrates the benefit of adaptive regional selection with negligible computational overhead. DAttention increased mAP50 and mAP50:95 by 0.3 percentage points, but also increased the parameter count and GFLOPs to 2.8 M and 6.5, respectively. This accuracy–cost trade-off is consistent with the additional computation required for deformable spatial sampling. EfficientHead improved mAP50 and mAP50:95 to 96.0% and 78.1%, respectively, while reducing the model to 2.3 M parameters and 5.1 GFLOPs and increasing FPS to 181.3.

The cumulative experiments further verify the complementary behavior of the modules. Combining C3k2_CFC and C2BRA increased mAP50 and mAP50:95 to 96.5% and 78.5%. After adding DAttention, the two metrics further increased to 96.9% and 79.2%, although the computational cost rose to 2.7 M parameters and 6.4 GFLOPs. Finally, EfficientHead compensated for this additional overhead. With all four modules enabled, ISD-YOLO achieved the best accuracy, with an mAP50 of 97.2% and an mAP50:95 of 79.8%, while using only 2.3 M parameters and 5.0 GFLOPs. Relative to the baseline, this corresponds to gains of 2.1 and 2.3 percentage points in mAP50 and mAP50:95, a reduction of 0.3 M parameters and 1.3 GFLOPs, and an increase of 24.6 FPS. These results indicate that the final architecture achieves a favorable accuracy–complexity trade-off: DAttention contributes spatial localization capability, whereas EfficientHead offsets its computational cost, allowing the complete model to remain lightweight and suitable for real-time seal detection.

**Fig 7:**
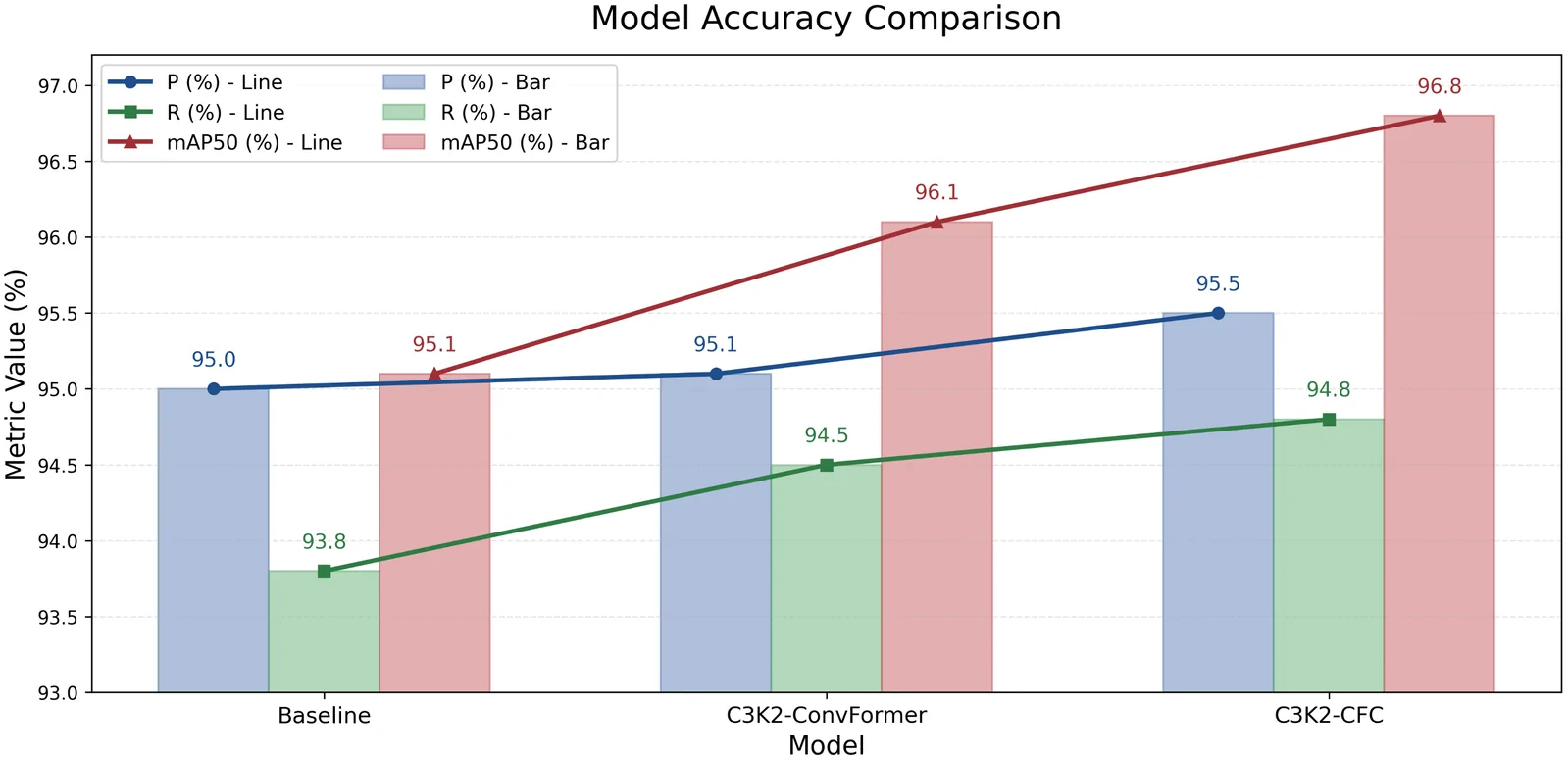
Ablation experiments on the constituent modules of C3k2_CFC.

As shown in Fig 7, a stepwise ablation study was further conducted on the key components of C3k2_CFC to verify the contribution of each substructure to detection performance. The experimental setup included three configurations: (1) Baseline, corresponding to the original C3K2 structure; (2) C3K2-ConvFormer, in which the ConvFormer module was introduced into C3K2; and (3) C3k2_CFC, in which CGLU was further incorporated on the basis of C3K2-ConvFormer.

As shown in Fig 7, the baseline model achieved P, R, and mAP50 values of 95.0%, 93.8%, and 95.1%, respectively. After ConvFormer was introduced into C3K2, model performance improved consistently, with mAP50 increasing to 96.1%, which represents a 1.0% improvement over the baseline. Meanwhile, P increased from 95.0% to 95.1%, and R rose from 93.8% to 94.5%, indicating that ConvFormer effectively enhanced feature representation and improved target recall. On this basis, CGLU was further incorporated to form C3K2_CFC, leading to continued performance gains. Specifically, mAP50 reached 96.8%, while P and R further increased to 95.5% and 94.8%, respectively. Such sequential enhancements illustrate that ConvFormer plus CGLU show robust synergy inside the C3K2 architecture and collectively enhance both framework accuracy and detection, thus confirming the utility of the suggested C3k2_CFC structure.

### 3.4 Comparative experiments

To further validate the performance advantages of the ISD-YOLO model, a series of comparative experiments were conducted on the seal dataset used in this study. The hardware configuration and training parameters used in these experiments were consistent with those described in Section 3.2.

**Fig 8:**
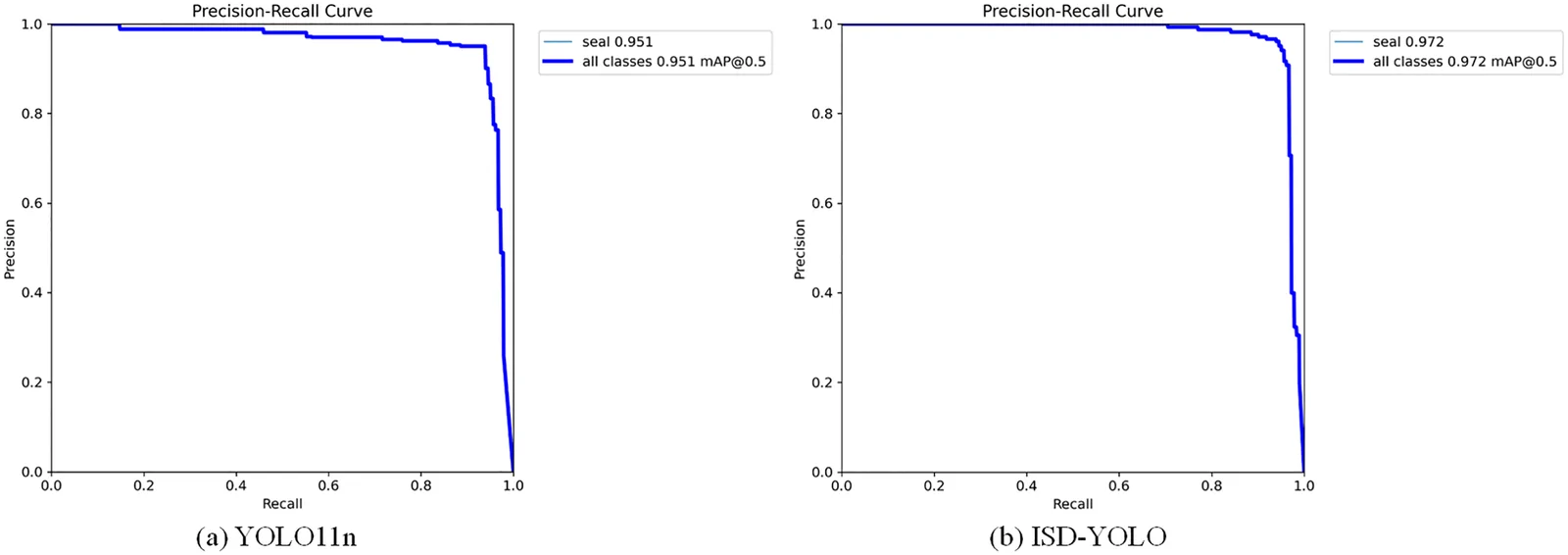
Comparison of mAP curves.

Fig 8 compares the P-R curves of the baseline model YOLO11n and the improved model ISD-YOLO on the test set. Both methods maintained high precision over most recall ranges; however, the P-R curve of ISD-YOLO consistently lay above that of YOLO11n, indicating higher precision at the same recall level and a slower performance decline in the high-recall region. Quantitatively, the baseline YOLO11n achieved an mAP50 of 0.951, whereas ISD-YOLO improved this value to 0.972. These results demonstrate that, compared with the baseline YOLO11n, the proposed ISD-YOLO delivers superior overall detection performance and greater robustness.

**Fig 9:**
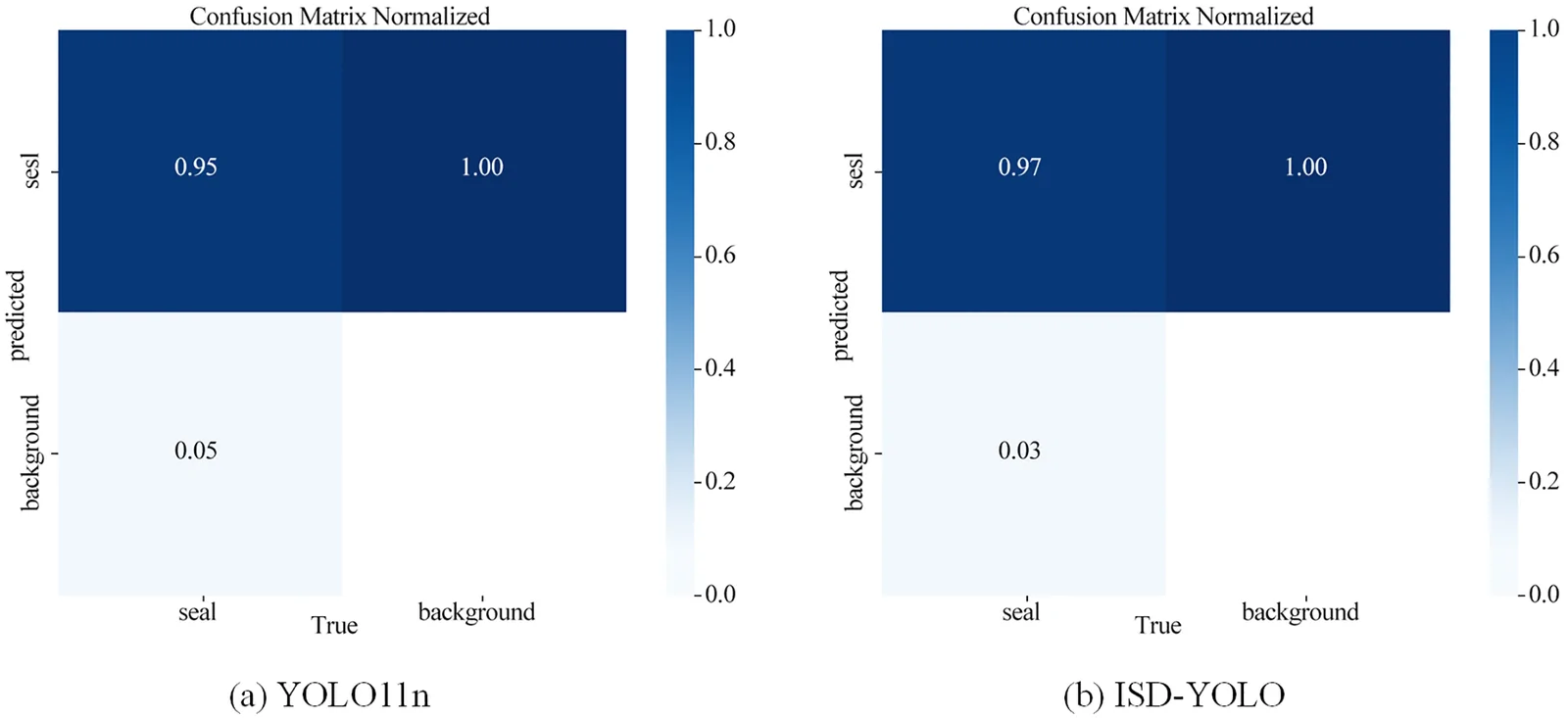
Comparison of confusion matrices on the test set.

As illustrated in Fig 9, a comparative evaluation was performed regarding the normalized confusion matrices of the fundamental model YOLO11n and the advanced model ISD-YOLO across the testing dataset. The diagonal entries of this matrix denote the fractions of accurate forecasts, while the off-diagonal entries represent errors. The findings indicate that both frameworks achieved a significant degree of precision in identifying the seal type. Nevertheless, when compared to the original YOLO11n, ISD-YOLO achieved a superior precise recognition ratio, which rose from 0.95 to 0.97. Such progress signifies a further strengthening in its capability to precisely detect the object class. Generally, the contrast of these confusion matrices proves that ISD-YOLO enhances distinction and identification quality for the seal type while preserving efficient background removal, thus providing more dependable detection outcomes.

Table 4 displays a comprehensive comparison regarding the experimental outcomes derived from various attention-driven feature refinement architectures. The fundamental structure, C2PSA, recorded an mAP50 value of 95.1%, an mAP50:95 ratio of 77.5%, and a processing velocity of 133.4 FPS. Conversely, C2DA raised the mAP50 metric to 95.6%, yet its mAP50:95 dropped to 76.8%, while the overall speed marginally decreased to 130.9 FPS. This outcome implies that its advancement in localization precision at high IoU levels was restricted. C2TSSA demonstrated a superior equilibrium between performance and speed, showing an mAP50 of 96.0%, an mAP50:95 of 77.6%, and a throughput of 176.3 FPS. This observation indicates that the framework improved feature abstraction while preserving substantial computational effectiveness. C2CGA reached the peak accuracy figures, with an mAP50 of 96.3% and an mAP50:95 of 78.0%. Nevertheless, its processing rate fell to 116.5 FPS, illustrating a relatively steep inference expenditure. On the contrary, the C2BRA mechanism utilized here reached an optimal integration of detection fidelity and live-time efficiency, exhibiting an mAP50 of 96.2%, an mAP50:95 of 77.8%, and a throughput of 161.7 FPS.

**Table 4:** Comparison of different attention-based feature enhancement modules.

Model	mAP50(%)	mAP50:95(%)	FPS
C2PSA	95.1	77.5	133.4
C2DA [40]	95.6	76.8	130.9
C2TSSA [41]	96.0	77.6	176.3
C2CGA [42]	96.3	78.0	116.5
Ours (C2BRA)	96.2	77.8	161.7

Table 5 contrasts model efficiency and intricacy among various detection components. The fundamental framework attained an mAP50 score of 95.1%, containing 2.6 M weights and 6.3 GFLOPs. Based on this framework, LQEHead enhanced mAP50 to 95.9% while decreasing the parameter count to 2.3 M and processing load to 6.1 GFLOPs, suggesting consistent advancements with a streamlined architecture. In contrast, MultiSEAMHead achieved the highest mAP50, reaching 96.2%, but its parameter count increased substantially to 4.5 M. Although its computational cost was only 6.0 GFLOPs, it still incurred a considerable increase in model size. SEAMHead achieved an mAP50 of 95.7%, with 2.4 M parameters and 5.8 GFLOPs, providing only limited accuracy gains despite lower model complexity. By comparison, the EfficientHead proposed in this study achieved the best computational efficiency while maintaining high accuracy. It reached an mAP50 of 96.0%, with 2.3 M parameters and a further reduction in computational cost to 5.1 GFLOPs. Overall, Table 4 shows that the proposed detection head substantially reduces computational overhead while maintaining competitive detection performance.

**Table 5:** Comparison of different detection heads.

Model	mAP50(%)	Params(M)	GFLOPS
Baseline	95.1	2.6	6.3
LQEHead [43]	95.9	2.3	6.1
MultiSEAMHead [44]	96.2	4.5	6.0
SEAMHead [45]	95.7	2.4	5.8
Ours (EfficientHead)	96.0	2.3	5.1

In addition, to further verify the advantages of the proposed algorithm, several mainstream target detection algorithms were selected for comparison with the ISD-YOLO model on the seal dataset constructed in this study. The results are presented in Table 6, where bold values denote the best performance for each metric.

**Table 6:** Comparison of results among different models.

Model	P(%)	R(%)	F1-score(%)	mAP50(%)	mAP50:95(%)	Params(M)	GFLOPS	FPS
YOLOv5n	92.8	91.0	91.9	94.5	77.3	2.2	5.8	145.0
YOLOX-n	92.8	91.7	92.2	95.4	78.9	2.9	10.7	106.7
YOLOv6n	92.6	96.2	94.4	96.8	80.2	4.2	11.5	139.3
YOLOv8n	96.8	93.4	95.1	96.0	79.4	2.7	6.8	147.0
YOLOv9t	95.4	91.2	93.3	95.6	79.1	1.7	6.4	137.1
YOLOv10n	93.4	92.3	92.9	95.2	77.6	2.3	6.5	174.0
YOLO11n	95.0	93.8	94.4	95.1	77.5	2.6	6.3	133.4
YOLOv12n	92.3	94.5	93.4	95.6	77.7	2.5	5.8	149.8
YOLOv13n	94.6	93.4	94.0	96.3	77.5	2.4	6.1	120.1
YOLO26n	87.7	89.1	88.4	92.9	74.2	2.4	5.2	187.1
Hyper-YOLO-t [46]	92.4	95.1	93.7	96.2	78.1	2.7	7.6	137.8
RT-DETR-l [35]	88.6	85.1	86.8	89.0	72.0	32.0	103.4	67.7
RTMDet-t [47]	92.8	93.4	93.1	95.6	79.2	4.6	13.4	117.9
ISD-YOLO	96.1	94.4	95.3	97.2	79.8	2.3	5.0	158.0

As shown in Table 6, ISD-YOLO achieves the highest F1-score and mAP50 among all compared models, reaching 95.3% and 97.2%, respectively. Although its precision and recall are not individually the highest, the leading F1-score indicates a better balance between false detections and missed detections. Its mAP50:95 reaches 79.8%, which is second only to the 80.2% achieved by YOLOv6n. Compared with the baseline YOLO11n, ISD-YOLO improves the F1-score, mAP50, and mAP50:95 by 0.9, 2.1, and 2.3 percentage points, respectively. Meanwhile, the parameter count decreases from 2.6 M to 2.3 M, the computational cost decreases from 6.3 to 5.0 GFLOPs, and the inference speed increases from 133.4 to 158.0 FPS. Although its parameter count and inference speed are not the best individual results, ISD-YOLO achieves the lowest GFLOPs and the third-highest FPS, indicating a favorable balance between detection accuracy and computational efficiency in the present experimental setting rather than a uniform advantage across all metrics.

From the perspective of feature fusion, conventional lightweight YOLO-series detectors generally rely on predetermined FPN- or PAN-style connections. Unidirectional FPN mainly transfers high-level semantics to shallow feature levels, whereas PAN introduces an additional bottom-up pathway but still uses fixed upsampling, downsampling, and concatenation operations without explicit content-dependent region selection. Transformer-based detectors can establish long-range feature interactions through attention, but the result of RT-DETR-l in Table 5 also illustrates the considerable model size and computational cost that may accompany this strategy in the present experimental setting. In contrast, ISD-YOLO combines efficient bidirectional cross-scale interaction with content-adaptive deep-feature enhancement. High-level semantic information is propagated toward the shallow detection levels, refined localization information is transferred back to the deeper levels, and C2BRA together with DAttention selectively emphasizes relevant regions and sampling locations before cross-scale fusion. This mechanism can reduce the propagation of responses produced by background text, table lines, and local occlusions while retaining complementary seal features at different scales.

Following the COCO area-based evaluation protocol, invoice seals were categorized as small (A≤32×32), medium (32×32≤A≤96×96), and large A≥96×96 targets. As shown in Table 7, ISD-YOLO achieves the highest mAP50_small_ and mAP50_large_ among all compared models, reaching 94.5% and 99.1%, respectively. Its mAP50_medium_ reaches 96.8%, tying for the best result with YOLO13n. Compared with the baseline YOLO11n, ISD-YOLO improves mAP50_small_, mAP50_medium_, and mAP50_large_ by 3.4, 2.3, and 1.1 percentage points, respectively. The more pronounced improvement for small seals demonstrates that ISD-YOLO can better preserve fine-grained features and detect challenging targets with limited pixels or blurred boundaries, while maintaining consistent performance across the evaluated target scales.

**Table 7:** Quantitative comparison of detection performance for invoice seals of different sizes.

Model	mAP50_small_	mAP50_medium_	mAP50_large_
YOLOv5n	90.2	94.0	97.7
YOLOX-n	92.0	95.5	98.1
YOLOv6n	93.2	96.6	98.7
YOLOv8n	91.8	95.2	98.6
YOLOv9t	91.7	95.1	98.6
YOLOv10n	90.9	94.3	98.9
YOLO11n	91.1	94.5	98.0
YOLOv12n	91.7	95.1	98.7
YOLOv13n	93.4	96.8	95.3
YOLO26n	91.3	94.6	98.0
Hyper-YOLO-t	92.5	95.9	98.7
RT-DETR-l	87.7	88.9	89.7
RTMDet-t	93.8	95.2	98.1
ISD-YOLO	94.5	96.8	99.1

**Fig 10:**
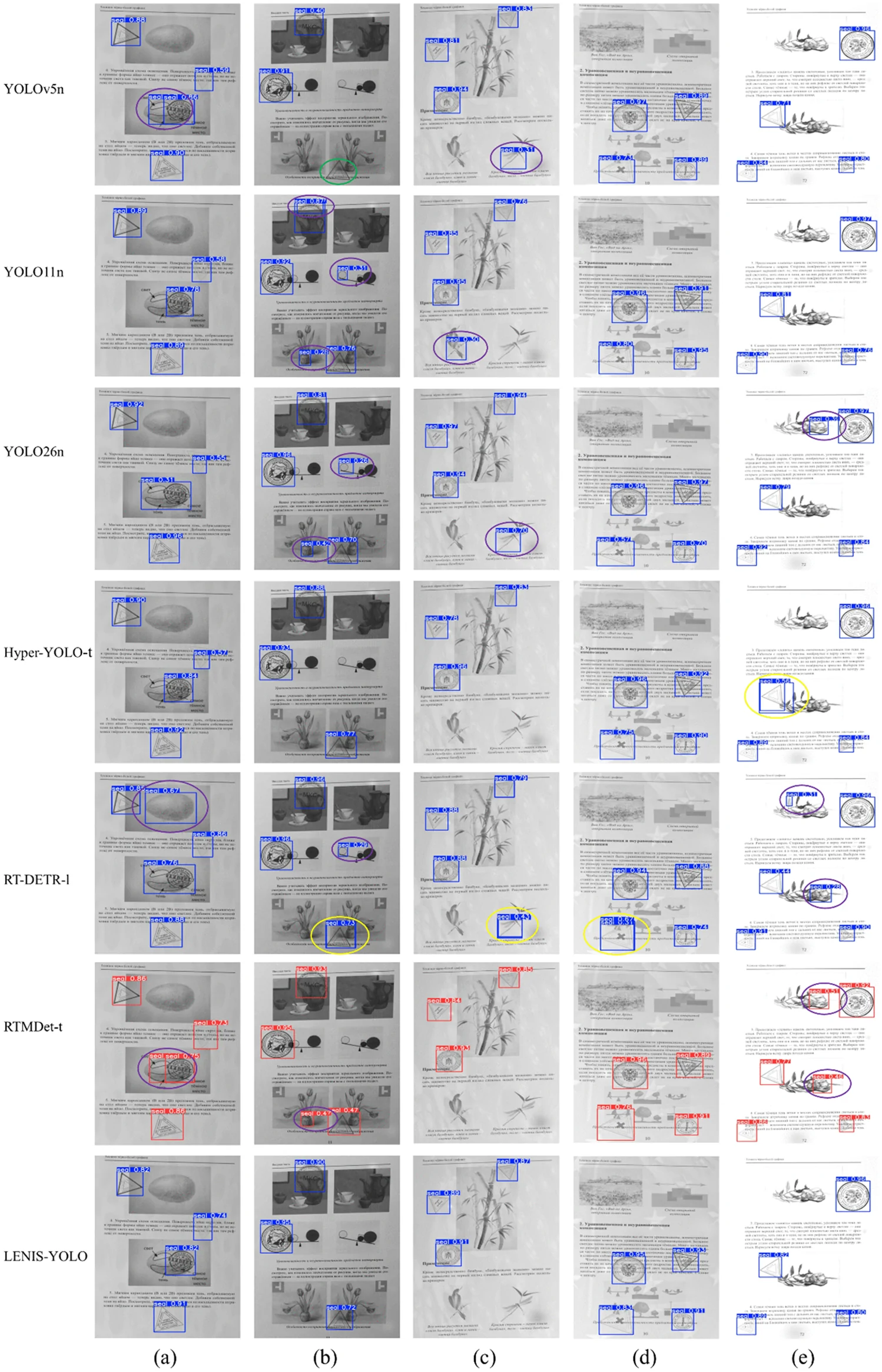
Comparison of seal detection results under complex background scenarios.

**Fig 11:**
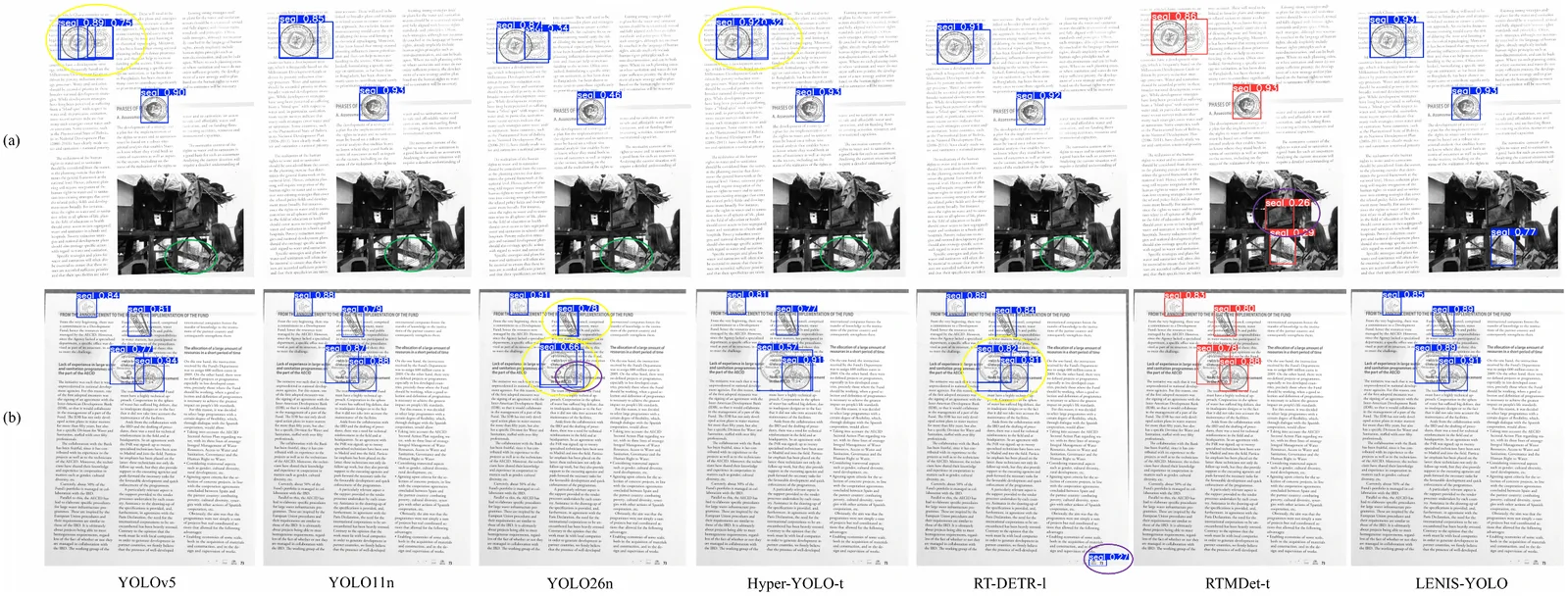
Comparison of seal detection results under partial occlusion scenarios.

Furthermore, to offer a more vivid assessment of the suggested approach, various top-tier detectors, such as YOLOv5, YOLO11n, YOLO26, Hyper-YOLO-t, RT-DETR-l, and RTMDet-t, were chosen for graphical analysis on the seal dataset utilized within this research. The experimental results are presented in Fig 10 and Fig 11. Specifically, Fig 10 presents comparative seal detection results in complex background scenarios, whereas Fig 11 shows the results in scenarios involving partial occlusion. As shown in Fig 10, under the different background conditions presented in Fig 10(a)-10(e), the ISD-YOLO algorithm accurately detects seals, with relatively few redundant bounding boxes, false detections, and missed detections. In contrast, YOLOv5, YOLO11n, YOLO26, Hyper-YOLO-t, RT-DETR-l, and RTMDet-t exhibit false detections, missed detections, and redundant bounding boxes to varying degrees, indicating favorable detection performance in the shown complex-background examples. Specifically, YOLOv5 produces false detections in Fig 10(a) and Fig 10(c), and a missed detection in Fig 10(b). YOLO11n produces false detections in Fig 10(b) and Fig 10(c). YOLO26 produces false detections in Fig 10(b), Fig 10(c), and Fig 10(e). Hyper-YOLO-t produces redundant bounding boxes in Fig 10(e). RT-DETR-l produces false detections in Fig 10(a), Fig 10(b), and Fig 10(e), and redundant bounding boxes in Fig 10(b), Fig 10(c), and Fig 10(d). RTMDet-t produces false detections in Fig 10(a), Fig 10(b), and Fig 10(e). Here, purple ellipses indicate false detections, green bounding boxes indicate missed detections, and yellow bounding boxes indicate redundant bounding boxes. As shown in Fig 11, when the seals are partially occluded, ISD-YOLO still accurately detects the target seals and achieves better overall detection performance than YOLOv5, YOLO11n, YOLO26, Hyper-YOLO-t, RT-DETR-l, and RTMDet-t. This suggests favorable detection performance in the shown partially occluded examples, whereas the other algorithms suffer from false detections, missed detections, and redundant bounding boxes. Specifically, as shown in Fig 11(a), YOLOv5, YOLO11n, YOLO26, Hyper-YOLO-t, and RT-DETR-l exhibit missed detections. As shown in Fig 11(b), YOLO26, RT-DETR-l, and RTMDet-t exhibit false detections. In addition, YOLOv5, YOLO26, RT-DETR-l, and Hyper-YOLO-t exhibit redundant bounding boxes. Here, purple ellipses indicate false detections, green bounding boxes indicate missed detections, and yellow bounding boxes indicate redundant bounding boxes. Overall, ISD-YOLO performs favorably in the evaluated complex-background and partial-occlusion examples, but broader robustness remains to be validated.

### 3.5 Actual detection results

**Fig 12:**
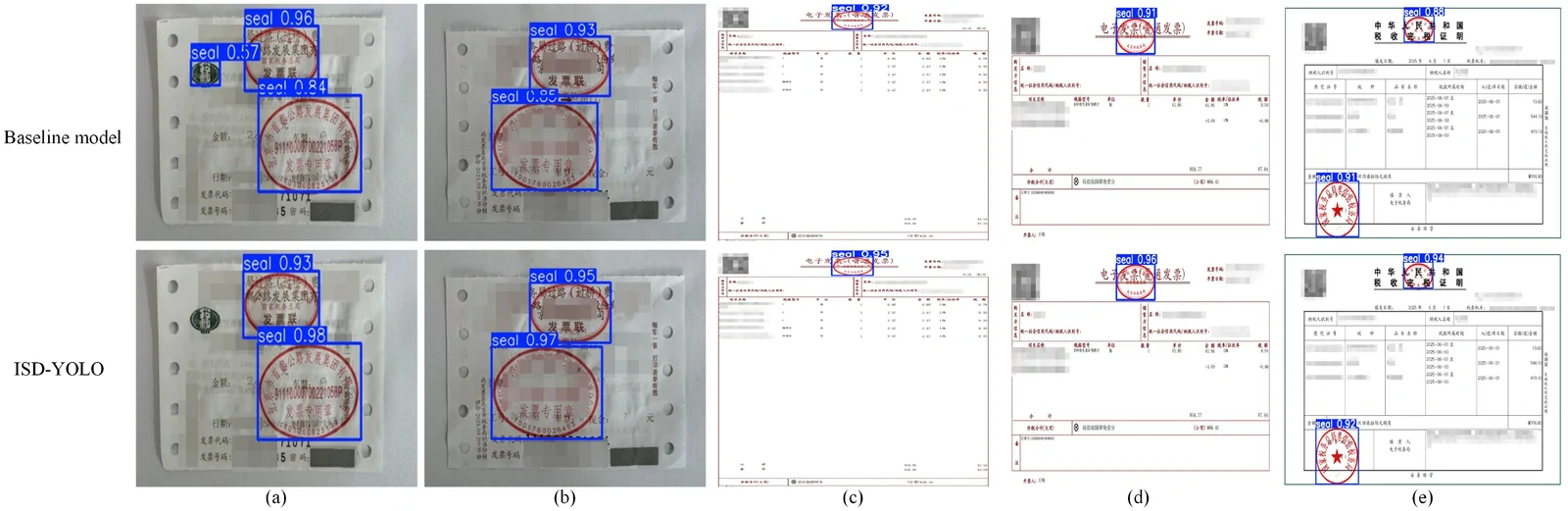
Comparison results with the baseline model on real invoice seal images.

To validate the practical effectiveness of ISD-YOLO for real invoice seal detection, multiple real invoice images were selected for visual evaluation, and the results are presented in Fig 12. As shown in Fig 12, the first row presents the detection results of the baseline model YOLO11n, whereas the second row shows those of the proposed ISD-YOLO model. For the same set of test samples, both algorithms detect seal targets in the invoices, but clear differences are observed in detection accuracy and stability. Specifically, the baseline model YOLO11n produces false detections in some samples by incorrectly identifying non-seal regions as seal targets, indicating insufficient discrimination between target regions and background information in real invoice scenarios. In contrast, ISD-YOLO yields more accurate results on the corresponding samples, effectively localizing seal regions in the invoices without obvious false detections. Moreover, its detection boxes align more closely with the target regions, demonstrating greater detection stability. These results indicate that the proposed ISD-YOLO model is robust and effective for real invoice seal detection and is better suited to the requirements of practical applications.

**Fig 13:**
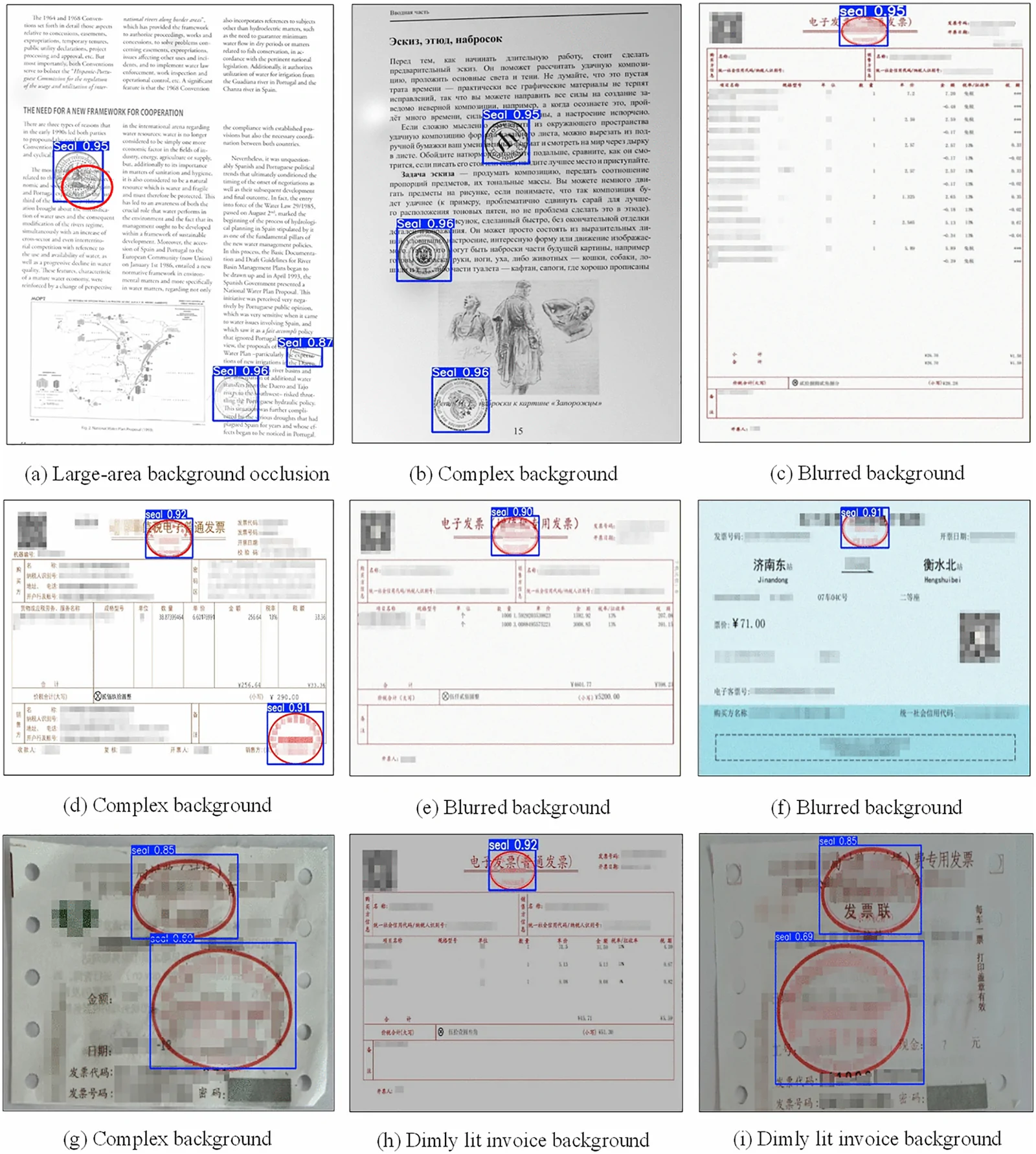
Invoice seal detection results under different scenarios.

To further evaluate the feasibility of ISD-YOLO for invoice seal detection in complex real-world scenarios, we selected real invoice images representing multiple degradation conditions for visual analysis. The results are shown in Fig 13. In the figure, bounding boxes denote the seal targets detected by the model, whereas red ellipses mark missed targets. As shown in Fig 13, ISD-YOLO accurately detected invoice seals against complex backgrounds, including interference from text, graphic patterns and tables, as well as under dimly lit and blurred background conditions. Overall, the model produced relatively few false positives and missed detections, suggesting a degree of adaptability to common background interference and image degradation in real-world settings. However, missed detections remained when the seals were extensively occluded, as indicated by the red ellipses in Fig 13. This may be because occlusion disrupts the complete contours and key texture information of the seals, making it difficult for the model to extract sufficient discriminative features. These visual results support the feasibility of ISD-YOLO for invoice seal detection under the evaluated real-invoice conditions, but do not establish robustness across all degradation scenarios.

To further clarify the applicability and limitations of ISD-YOLO in practical settings, Table 8 summarizes typical challenging conditions, potential detection errors, and their causes based on the size-stratified detection results presented in Table 7 and the visual results shown in Figs 10-13. It should be noted that the available annotation files contain only seal class labels and bounding-box information; attributes such as occlusion level, color fading, image blur, rotation angle, and background complexity were not annotated separately. Therefore, apart from the size-stratified results reported in Table 7, Table 8 primarily provides a qualitative analysis of typical errors and their causes. Error counts, error proportions, and separate mAP results for individual conditions are not reported because the necessary scene-level annotations are unavailable.

**Table 8:** Detection performance and potential errors of ISD-YOLO under challenging conditions.

Challenging Condition or Error Type	Possible Detection Outcome	Cause Analysis	Current Evidence
Seals of different sizes	Small seals are more susceptible to insufficient feature representation or localization errors	Small targets contain fewer pixels and provide limited edge and internal texture information	Table 6 provides size-stratified mAP50 results, although the size-classification criteria require further verification
Extensive occlusion	Missed detections	Occlusion disrupts the complete contours and key textures of seals, resulting in insufficient discriminative information	Missed-detection cases are observed in Fig 13
Image blur or seal fading	Reduced detection confidence, missed detections, or bounding-box shifts	The edge, color, and texture features of seals are weakened	Sample-level qualitative analysis was conducted, but no separate statistical analysis was performed
Seal rotation	Possible localization errors or missed detections	Changes in orientation and geometric structure cause shifts in the feature distribution	Rotation attributes were not independently annotated, and no separate statistical results are available
Red text, logos, or seal-like graphics	Potential false detections	Their colors, local shapes, or textures may resemble those of seals	These elements are discussed only as potential sources of interference and were not counted separately
Text overlap, table lines, and complex backgrounds	False detections, duplicate detections, or bounding-box localization errors	Background lines and textual structures interfere with seal contours and internal texture features	Figs 10–13 provide qualitative results, but no corresponding condition-specific mAP results are reported

Overall, these results indicate that ISD-YOLO exhibits a degree of adaptability to seals of different sizes, as well as to complex backgrounds, dim conditions, and image blur. However, missed detections may still occur under extreme conditions such as extensive occlusion. Red text, logos, table lines, and other graphical elements with colors or shapes similar to those of seals may interfere with the model’s discrimination, resulting in false detections, duplicate detections, or bounding-box localization errors. Future research will further expand the dataset to include more seal types and invoice seal images from a wider range of application scenarios, while continuously optimizing the ISD-YOLO model. In addition, test samples will be stratified according to occlusion severity, fading severity, blur severity, rotation angle, and background complexity to further assess the model’s detection performance and robustness under different complex conditions.

### 3.6 Limitations and future work

Although ISD-YOLO achieved favorable performance in the seal detection task, several limitations remain. The current dataset provides limited coverage of seal categories and complex degradation scenarios, and the model may still miss seals under extensive occlusion or substantial degradation in image quality. In this study, the training, validation, and test sets were constructed using image-level random splitting, without grouping the images according to document sources, invoice layouts, or related factors. In addition, near-duplicate invoices and images with highly similar layouts were not specifically identified and removed. Consequently, samples with similar visual characteristics may be distributed across different subsets, which limits the assessment of the model’s generalization across invoice layouts and data sources. Model training also remains substantially dependent on large-scale manually annotated data, leaving room to reduce annotation costs and improve training efficiency. Furthermore, deployment tests have not yet been conducted on actual low-power platforms or edge devices. Consequently, metrics such as inference latency, memory usage, and model storage overhead on real devices require further validation. The present study focuses primarily on invoice-seal detection and localization and does not address subsequent tasks such as seal-character recognition, authenticity verification, or invoice-text recognition.

To address these limitations, future work will focus on the following aspects: (1) expanding the dataset in terms of both scale and sample diversity by adding seal data from different categories and samples involving complex degradation scenarios, including uneven illumination, image blur, watermark overlap, and extensive occlusion. In addition, document-source and invoice-layout information will be further organized, near-duplicate samples will be removed, and the training, validation, and test sets will be divided according to document, layout, or data source. An independent external test set will then be established. On this basis, ISD-YOLO will be further optimized to improve its robustness in complex real-world scenarios and its generalization across different invoice layouts and data sources; (2) investigating unsupervised or weakly supervised learning strategies to reduce the model’s dependence on large-scale annotated data, lower annotation costs, and improve training efficiency; (3) investigating seal-character recognition and integrating it with seal-authenticity verification and invoice-text recognition to develop an end-to-end system for intelligent invoice auditing, thereby improving the practical applicability of the model; and (4) conducting deployment tests on low-power platforms or edge devices and evaluating inference latency, memory usage, and model size to validate the edge-deployment performance of ISD-YOLO.

## 4. Conclusion

This study presents ISD-YOLO, a lightweight invoice seal detection algorithm developed on the basis of YOLO11n to improve detection accuracy while reducing computational cost. Within ISD-YOLO, the C3k2_CFC module is first introduced by combining the local feature enhancement capability of ConvFormer with the gated feature selection mechanism of CGLU, thereby strengthening the model’s fine-grained representation of multiscale features, including the overall seal structure, edge details, and internal text. The C2BRA module is then incorporated into the deeper layers of the backbone network, where a two-stage routing strategy integrating coarse-grained and fine-grained selection dynamically identifies key feature regions, reducing computational redundancy while improving sensitivity to seal shape, edges, and internal textures. Building on this design, the DAttention module is further employed to enhance attention to critical regions and effectively mitigate interference from complex backgrounds. Finally, a lightweight detection head, EfficientHead, is constructed to reduce model complexity and improve inference efficiency, thereby enhancing the potential for edge deployment. This framework utilizes a dual-branch parallel structure and employs group convolution to facilitate effective extraction of multi-scale seal characteristics, allowing for simultaneous refinement of classification and localization tasks while minimizing both model parameters and processing load. Experimental results show that, compared with the original model, ISD-YOLO enhances mAP50 and mAP50:95 by 2.1% and 2.3%, respectively. Compared with several advanced detection algorithms, ISD-YOLO achieves an F1-score of 95.3% and an mAP50 of 97.2%, with the highest F1-score and mAP50 among the compared methods, while its mAP50:95 is not the highest; these results indicate a favorable balance between detection accuracy and computational efficiency rather than an advantage across every metric. Simultaneously, ISD-YOLO incorporates merely 2.3 M parameters and necessitates only 5.0 GFLOPs, thus substantially decreasing model intricacy and operational overhead while maintaining detection precision. Furthermore, the ablation analysis additionally verifies the beneficial impact of every component on performance enhancement. Overall, the results support the effectiveness of ISD-YOLO for invoice seal detection under the evaluated conditions, while its generalization to broader degradation scenarios and its deployment on actual edge devices require further validation.
